# Structure and properties of Sn-Cu lead-free solders in electronics packaging

**DOI:** 10.1080/14686996.2019.1591168

**Published:** 2019-03-08

**Authors:** Meng Zhao, Liang Zhang, Zhi-Quan Liu, Ming-Yue Xiong, Lei Sun

**Affiliations:** a School of Mechatronic Engineering, Jiangsu Normal University, Xuzhou, China; b Institute of Metal Research, Chinese Academy of Sciences, Shenyang, China; c College of Mechanical & Electrical Engineering, Nanjing University of Aeronautics and Astronautics, Nanjing, China

**Keywords:** Sn-Cu, microstructures, IMC, mechanical properties, 40 Optical, magnetic and electronic device materials, 100 Materials, 103 Composites, 106 Metallic materials, 503 TEM, STEM, SEM

## Abstract

With the development of lead-free solders in electronic packaging, Sn-Cu lead-free solder has attracted wide attention due to its excellent comprehensive performance and low cost. In this article, we present recent developments in Sn-Cu lead-free solder alloys. From the microstructure and interfacial structure, the evolution law of the internal structure of solder alloy/solder joint was analysed, and the model and theory describing the formation/growth mechanism of interfacial IMC were introduced. In addition, the effects of alloying, particle strengthening and process methods on the properties of Sn-Cu lead-free solders, including wettability, melting and mechanical properties, were described. Finally, we outline the issues that need to be resolved in the future research.

## Graphical abstract

**Figure:**
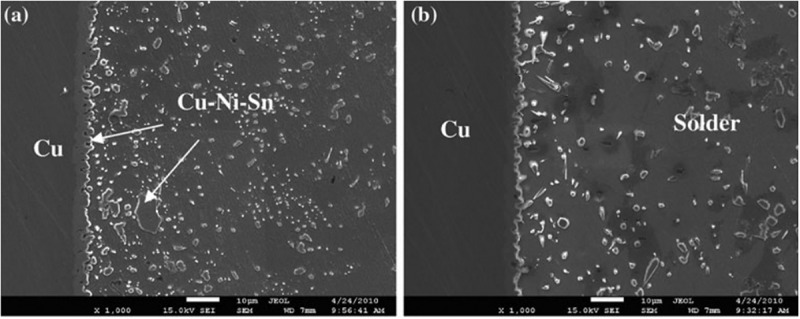

This figure presents the interface morphology of Sn-Cu-Ni-xPr joint. (a) x = 0, (b) x = 0.05wt%. It was clear that with the addition of 0.05% Pr, the thickness of IMC layer decreases significantly and the formation of voids at the interface is inhibited.

## 1. Introduction

In the electronics industry, solder joints connect electronic components with the printed circuit boards [1]. Traditional Sn-Pb solder was widely used in the electronic packaging industry because of its performance advantages. However, with the increasing awareness of environmental protection, the toxicity of lead has attracted much attention. Japan, EU and other countries and regions have promulgated laws to gradually prohibit the use of lead in the electronic packaging [2–7]. These initiatives provide a good opportunity for the development of new lead-free solder. Hence Sn-base lead-free solders, including Sn-Ag [8], Sn-Bi [9], Sn-Cu [10], Sn-In [11], Sn-Sb [12] and Sn-Zn [13] have become the potential candidates for Sn-Pb solder replacement.

Compared with traditional Sn-Pb solder, Sn-Cu solders exhibit the higher melting point [14–18]. However, there is still potential for the further development of Sn-Cu solder alloys with higher properties. The eutectic Sn-0.7Cu alloys have become the focus of research, since the eutectic alloys have the advantages of low melting point, narrow crystallization temperature range, good fluidity and low tendency of hot cracking and segregation. Among the new Pb-free alloy candidates, Sn-0.7Cu solders are inexpensive and exhibit promising characteristics [19]. As the practicability of Sn-Cu solder has been confirmed continuously, Sn-0.7Cu solder has been widely used in wave soldering process [20,21]. In order to research several properties of Sn-Cu solder alloys, a lot of researches have been done, including the structure/interface evolution, the wettability and mechanical properties, by means of alloying, particle strengthening and solidification rate control.

This article reviews the research progress of Sn-Cu solder alloys. The microstructure and interfacial evolution of solder alloy were summarized. The wettability, mechanical properties and melting properties of Sn-Cu lead-free solders were analysed. Combined with alloying and particle strengthening methods, the research results of modification of solders were discussed.

## 2. Microstructure

**Figure 1.**
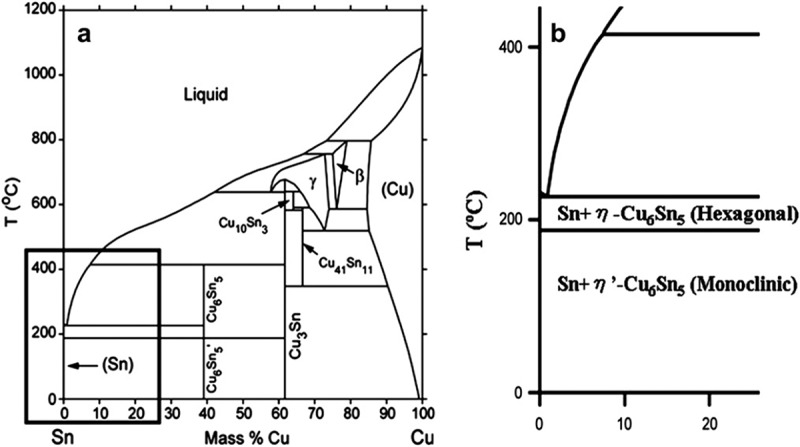
(a) Sn–Cu phase diagram and (b) magnification of the Sn-rich corner of (a). Reproduced with permisssion from [22] (a) and [23] (b).

The research of microstructure is vital for evaluating lead-free solder since the microstructure of solder directly determines the properties of the solder and the solder joints. According to the phase diagram (Figure 1 [22,23]), the stable intermetallic phases below 300 °C are Cu_3_Sn and Cu_6_Sn_5_ phases [24]. Sn-0.7Cu is near eutectic system and the main phases are β-Sn and Cu_6_Sn_5_ at room temperature [25]. The eutectic reaction of L→β-Sn + η-Cu_6_Sn_5_ exists in Sn-0.7Cu at 227 °C. With the further cooling of the melt to 186 °C, the transformation of the Cu_6_Sn_5_ phase occurs from the hexagonal η-Cu_6_Sn_5_ to a monoclinic polymorph η’-Cu_6_Sn_5_ [26–29]. This solid phase transformation has a significant impact on solder joint reliability [23,30].

The effect of Cu content on the microstructure of Sn-xCu was investigated by Hung et al. [31]. It was found that the addition of Cu played a refining role on the β-Sn. Cu_6_Sn_5_ increased with the increase of Cu content. For Sn-1.3Cu and Sn-1.7Cu samples, a large number of Cu_6_Sn_5_ particles distribute evenly in the matrix and play a role of particle strengthening. However, with the increase of Cu content, the melting temperature of the solder alloy will increase significantly, which will directly affect the soldering temperature.

The remarkable effect of element doping on the equilibrium system of alloys, such as phase transformation and microstructural stability, has been widely confirmed [26,32–36]. According to the solubility of alloying elements in Cu_6_Sn_5_, the doped elements can be divided into two categories: (i) Ni, Au, Sb, In, Co, Pt, Pd and Zn are easily dissolved in Cu_6_Sn_5_ intermetallic compounds (IMCs); and (ii) elements (Bi, Ag, Fe, Al, P, rare-earth elements, Ti and S) have poor solubility in the Cu_6_Sn_5_ intermetallic [37].

**Figure 2.**
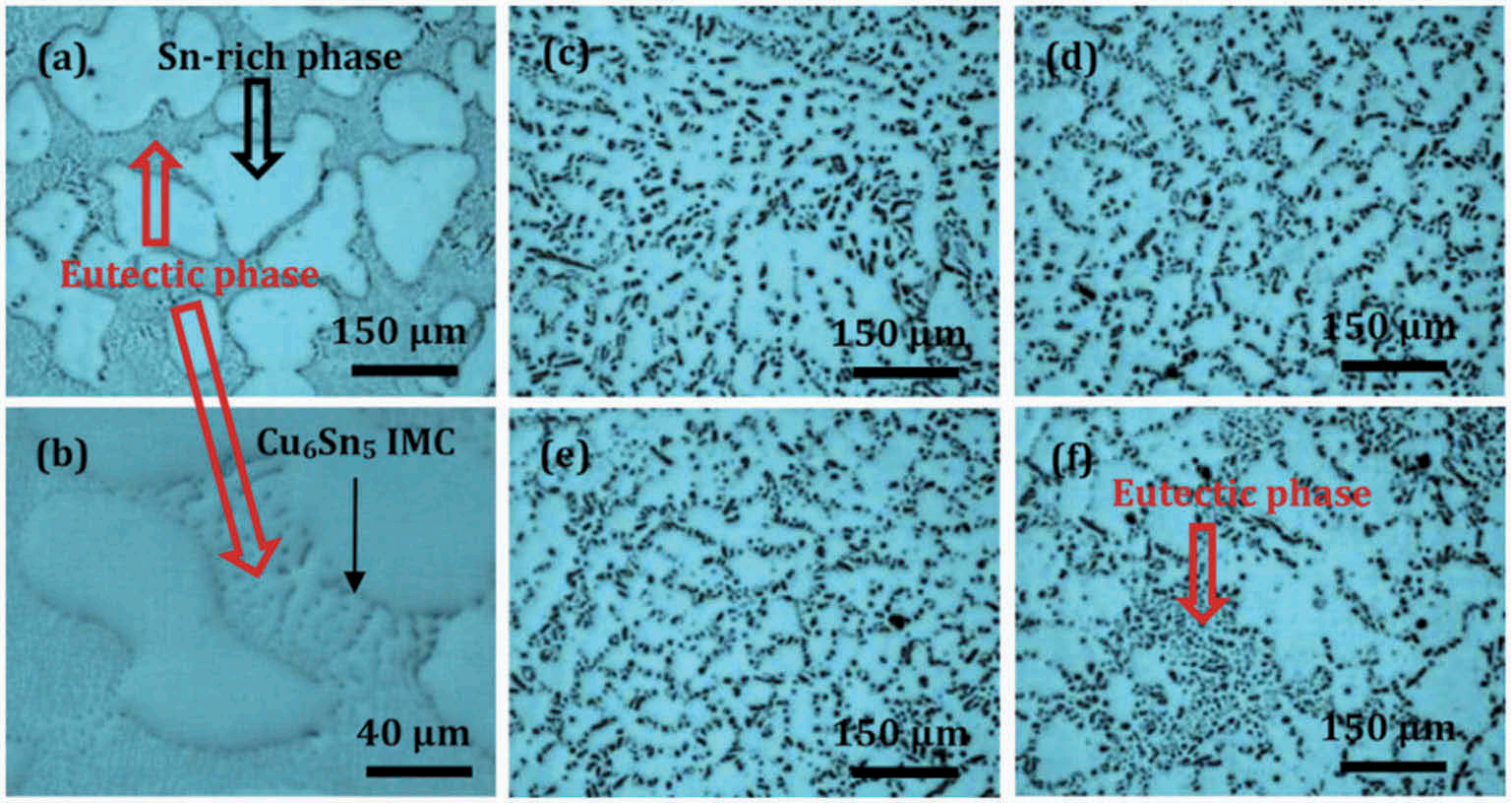
Optical microstructures of Sn-0.7Cu-xAl (x = 0–0.075) lead-free solder alloys (a) x = 0, (b) magnified image of eutectic in (a), (c) x = 0.01, (d) x = 0.025, (e) x = 0.05, and (f) x = 0.075. Reproduced with permission from [39].

Al is often used in the study of lead-free solder because of its low density, good electroconductivity and thermal conductivity. It has been reported that the addition of Al in hypereutectic Sn-Cu solder alloys resulted in the remarkable refinement of primary Cu_6_Sn_5_ grains since the introduction of δ-Cu_33_Al_17_ or γ-Cu_9_A_l4_ with the significant undercooling structure before Cu_6_Sn_5_ grain growth leads to the heterogeneous nucleation of Cu_6_Sn_5_ [38]. Figure 2 depicts the microstructure of Sn-0.7Cu-xAl (x = 0 ~ 0.075) solder alloy [39]. The microstructures of Sn0.7Cu solder alloys are composed of β-Sn and Cu_6_Sn_5_/β-Sn eutectic composition, while the solidified Sn-0.7Cu-0.075Al alloy is composed of β-Sn, Cu_6_Sn_5_ and δ-Al_2_Cu [40]. The Al addition makes the microstructures of the composite solder finer and the dispersion of Cu_6_Sn_5_ IMC in the matrix of β-Sn as shown in Figure 2(c–f), which is caused by Al atoms dissolution into β-Sn and eutectic refinement. In addition, with the increase of Al concentration the volume fraction of eutectic phase increases and Cu atoms and Al atoms tend to segregate in a certain region. After the precipitation of Cu_6_Sn_5_ IMC during solidification, Al_2_Cu phase was formed by the reaction of Cu_6_Sn_5_ IMC and Al, which could inhibit the formation of Cu_6_Sn_5_ phase.

**Figure 3.**
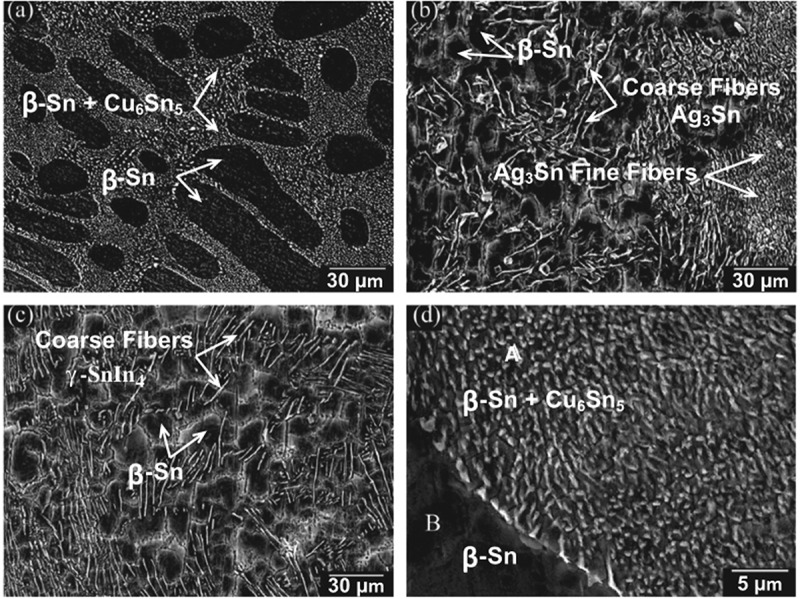
Microstructure of as-solidified: (a) Sn-0.7Cu, (b) Sn-0.7Cu-2Ag and (c) Sn-0.7Cu-2In solder alloys, with energy-dispersive spectroscopy analysis of selected areas in the Sn–0.7Cu solder: (d) SEM image of selected area. Reproduced with permission from [43].

The addition of appropriate amount of Ag to Sn-Cu solder alloy can promote the formation of network eutectic structure and heterogeneous nucleation reaction of β-Sn/Cu_6_Sn_5_ and refine the grain size of β-Sn [41,42]. This change is mainly caused by the formation of Ag_3_Sn in the solder metal matrix. Figure 3 shows a typical scanning electron microscopy (SEM) micrograph of as-solidified Sn-0.7Cu solder alloy and the solidification structure containing Ag or In addition [43]. The average diameters of β-Sn grain and Cu_6_Sn_5_ particles are 20–70 μm and 0.5–0.8 μm, respectively (as shown in Figure 3(a,d)). With Ag addition, a larger number and smaller size of IMC particles (Ag_3_Sn and Cu_6_Sn_5_) are observed. In Sn-0.7Cu-2Ag solder, the number of Ag_3_Sn particles is more than that of Cu_6_Sn_5_ particles due to the influence of interface energy [44]. Similarly, for Sn-0.7Cu-2In solder, γ-SnIn_4_ IMC is found, which promoted the formation of eutectic regions on the surface of β-Sn matrix and inhibited the formation of large β-Sn dendrite cells. On the other hand, the addition of highly active In resulted in the formation of γ-SnIn_4_ IMC, which reduced the activity of Sn, and ultimately inhibited the formation of Cu_6_Sn_5_ IMC.

Zhu et al. [45] added micron-sized Zn particles to Sn-0.7Cu solder, and found that with the increase of Zn content, β-Sn refined continuously. This is because the addition of Zn increased the number of nucleation particles, resulting in the grain size of the primary crystal of β-Sn gradually reduced, inhibiting the growth of β-Sn phase. But when the content reached 0.2%, β-Sn phase tended to coarsen. The shear strength of solder was improved to a certain extent because the undercooling of solder was restrained and the microstructure was refined, which strengthened the blocking effect of grain boundary on dislocation. The formation of Cu_5_Zn_8_ particles caused by Zn addition trapped in the Cu_6_Sn_5_ grain boundary and played a dispersion-strengthening role [46]. The author held that the addition of Zn should not be excessive. From the microstructure of Sn-0.7Cu-1.0Zn, the size of Cu_5_Zn_8_ particles began to show coarsening. If further increase the Zn addition, the size of Cu_5_Zn_8_ particles in the microstructure will be further increased, and the properties of the solder will deteriorate because Zn is easy to be oxidized.

As a metal element adjacent to the Cu element, Ni has the same atomic radius and similar structure and properties. Therefore, Ni is also used as the addition element in the modification of Sn-Cu alloy. In general, the Cu atom in the solder alloy can be replaced by nano-Ni particles, and a small amount of dispersed (Cu,Ni)_6_Sn_5_ phase can be formed in the primary phase and network eutectic structure, which can refine the primary β-Sn phase. A cellular-type eutectic microstructure was observed in Sn-0.7Cu-0.05Ni solder casting [47]. With the increase of Ni content (0–0.1wt.%), the volume fraction of primary β-Sn decreased gradually so that the more eutectic structure was observed in the microstructure of Sn-0.7Cu-xNi solder [48]. In addition, a microstructural feature named ‘coral zones’ was observed in the Ni-containing alloys, which contain significantly smaller intermetallics. While the formation mechanism of the coral zones is not yet clear.

Ge et al. [49] found that Fe particles addition can effectively improve the microstructure of Sn-0.7Cu solder matrix. With the addition of 0.025% Fe particles, the Cu_6_Sn_5_ grains inside the solder appear as needle in the β-Sn matrix. When the content of Fe particles reaches 0.05%, the shape of Cu_6_Sn_5_ changed and distributed in the β-Sn matrix. With the further increase of Fe, the microstructure of the solder alloy has coarsened. This happened mostly because Fe easily reacts with Sn forming FeSn_2_ phase, which changes the original microstructure balance of the solder. Tsao et al. [50] reported the effect of nano-TiO_2_ particles addition on the microstructure of Sn-0.7Cu solder. Adding appropriate amount of TiO_2_ particles can effectively refine the β-Sn matrix, inhibit the growth of Cu_6_Sn_5_ grains and advance the fine Cu_6_Sn_5_ grains dispersing in the β-Sn matrix. According to Table 1, with the increase of TiO_2_ content, the average grains size of both Cu_6_Sn_5_ and β-Sn decrease significantly. The decrease of average spacing of Cu_6_Sn_5_ IMC means that the number of Cu_6_Sn_5_ IMC increases. In addition, the eutectic area is increased by 125% (30 ± 7% of Sn0.7Cu solder vs. 68 ± 7% of Sn0.7Cu-1TiO_2_ solder).

**Table 1.** Phase constituents, average grain size and average spacing of the composite solder [50].

Sample	TiO_2_ addition (wt.%)	Cu_6_Sn_5 _(μm)		β-Sn (μm)	Eutectic area (vol.%)
		Average size	Average spacing		
Sn-0.7Cu	0	2.3 ± 0.9	1.9 ± 0.46	29 ± 4	30 ± 6%
1	0.25	0.4 ± 0.2	1.2 ± 0.36	23 ± 5	45 ± 6%
2	0.5	0.24 ± 0.06	0.20 ± 0.08	20 ± 4	42 ± 9%
3	1	0.18 ± 0.05	0.17 ± 0.09	16 ± 4	68 ± 7%

**Figure 4.**
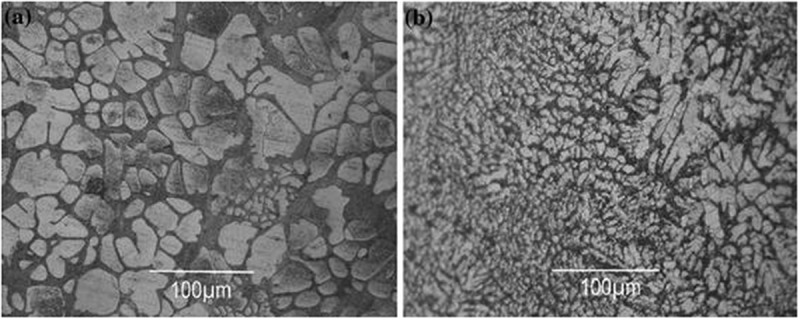
Optical micrographs for (a) Sn-0.7%Cu, (b) Sn-0.7%Cu-0.5%RE. Reproduced with permission from [52].

Rare earth (RE), and especially lanthanide elements are invaluable in the development of new lead-free solders, and have attracted attention of many researchers [51]. The effect of Ce and La on the microstructure and mechanical properties of Sn-0.7Cu alloy were researched [52]. When the addition of RE reaches 0.5%, β-Sn phase is refined obviously. The radial grain size is reduced from 30–50 μm to 5–10 μm, and the IMC Cu_6_Sn_5_ is fine and uniformly distributed in the β-Sn matrix, as shown in Figure 4.

**Figure 5.**
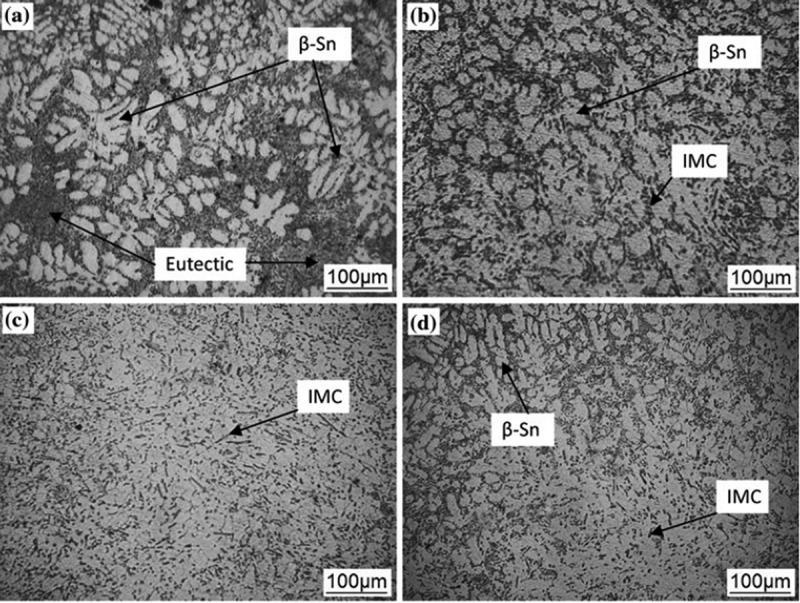
Optical microstructure for Sn-Cu-Ni-xPr solder alloy (a) x = 0, (b) x = 0.03, (c) x = 0.05, (d) x = 0.08. Reproduced with permission from [53].

The effect of Pr on the microstructures of Sn-Cu-Ni solders was investigated [53]. Figure 5 shows the matrix β-Sn and IMC phases (Cu_6_Sn_5_, (Cu, Ni)_6_Sn_5_) distributing between the β-Sn grain boundaries in microstructure of as cast alloy. The grain size of β-Sn is refined and the structure of solder alloy exhibits more uniform. When Pr content is 0.05%, the alloy shows favourable properties, which may be due to the refining influence of rare earth elements on the solder matrix and the nucleation and growth of PrSn_3_ IMC.

Similar to Pr, the addition of Nd can effectively refine the matrix of Sn-Cu-(Ni) solder [54]. As Pr and Nd have the surface activity, they tend to aggregate at the frontier of solidification interface during the solidification process, which increases the composition of solder alloy supercooling, and some crystals appear cystiform growth instead of the original flat growth pattern, reducing the dendrite spacing and refining the matrix structure. Rare earth elements have obvious grain boundary segregation effect. When the impurity element adsorbed on the grain boundary, the energy of grain boundary is decreased, which reduces the driving force of interface movement and impedes grain boundary movement and limits the growth of grain in solder matrix. On the other hand, the proper amount rare earth elements can significantly improve the microstructure and properties of the solder, but excessive addition will precipitate a large number of rare earth rich phases in the solder, which is easy to be oxidized and lead to the tin whisker problem of solder.

**Figure 6.**
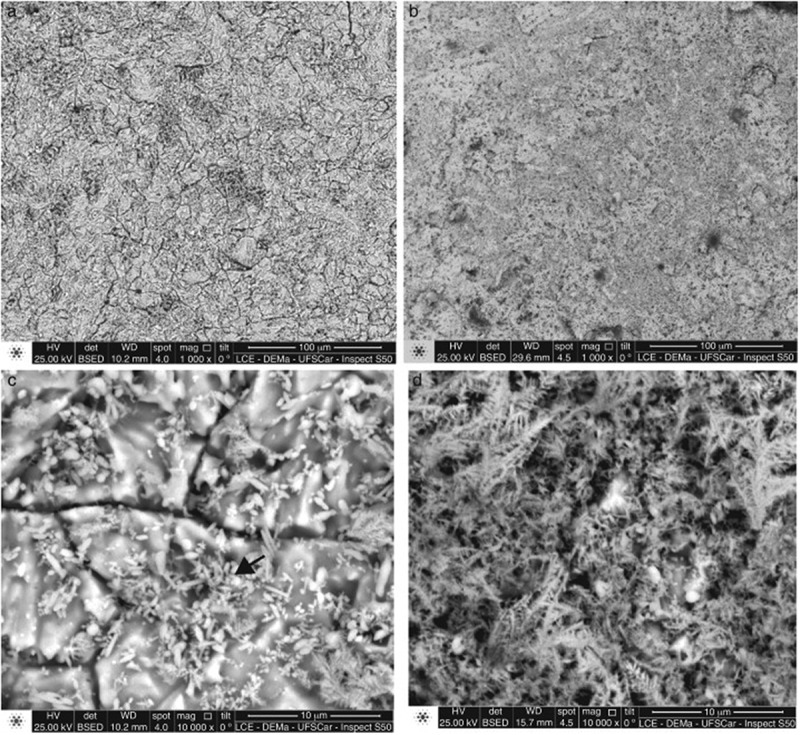
SEM (BSE) microstructures of the Sn–0.7Cu alloy droplet with size of 125–150 μm showing (a and c) features concerning hot extruded and (b and d) cold extruded specimens. Reproduced with permission from [59].

Attempting to study the processing technology on the basis of existing research may change some properties of the alloy [55–57], for example, the high performance can be obtained by powder metallurgy technology and the samples extruded at high temperature exhibit better microscopic properties [58]. Figure 6 shows a typical microstructure of as-extruded Sn-0.7Cu alloys [59]. The microstructure obtained by cold extrusion is characterized by finer grains and lower porosity. It seems that the extrusion process promotes profound changes in the microstructural characteristics of solder since the eutectic structure has not been found in the samples. However, the microstructures of cold extruded samples seem to retain some characteristics during atomization, such as dendritic IMC particles with good distribution of Cu_6_Sn_5_ IMC (Figure 6(c,d)), rod-like or fine alveolar, as marked by the arrow, which may lead to mechanical strength related results.

In addition, the rapid and directional solidification solder alloys have finer microstructures with less microsegregation, which ultimately improves the mechanical properties of solder joints [60]. Alternating lamellar eutectic structure and Sn-rich phase were observed in the longitudinal section of directionally solidified hypereutectic Sn-1.0Cu solder, growing in a specific direction, which results in the anisotropic characteristics of the directionally solidified solder because the compressive strength along the longitudinal direction is much higher than that along the transverse direction [61]. The large dynamic supercooling occurring under rapid solidification is the intrinsic mechanism of such a layered structure.

The Cu_6_Sn_5_ single fibre spacing and single crystal diameter are mainly controlled by solidification rate during solidification, and both of them decrease with the increase of the solidification rate [62]. In addition, the melt overheating treatment on Sn-Cu solder alloy can obviously refine the solidified microstructure of the alloy, make the interfacial compound layer of the soldering joints thinner, and improve the roughness of the interface. After structural transformation a large number of solid-like clusters are broken, and the clusters which could become the critical nucleus directly disappear or decrease. The smaller clusters can reach the critical nucleation size only at a greater degree of supercooling. With the increase of degree of supercooling, the nucleation rate increases and the critical nucleation radius decreases, which leads to the refinement of solidification structure.

## 3. Interfacial reaction

For lead-free solders, the metallurgical reaction between solder and substrate forms IMCs during soldering. IMCs such as Cu_6_Sn_5_ and Cu_3_Sn are often formed at the solder/substrate interface in the soldering joints [63]. With aging, IMCs grow at the interface and may be accompanied by the formation of Kirkendall voids. The rapid growth of brittle IMCs and formation of voids at the interface will worsen the performance of soldering joints. Therefore, controlling the thickness of IMCs and voids at the interface can effectively improve the reliability of soldering joints. At present, the researches on inhibition of IMCs at the Sn-Cu lead-free solder interface mainly focus on alloying, particle strengthening, soldering process optimization and substrate and coating material.

**Figure 7.**
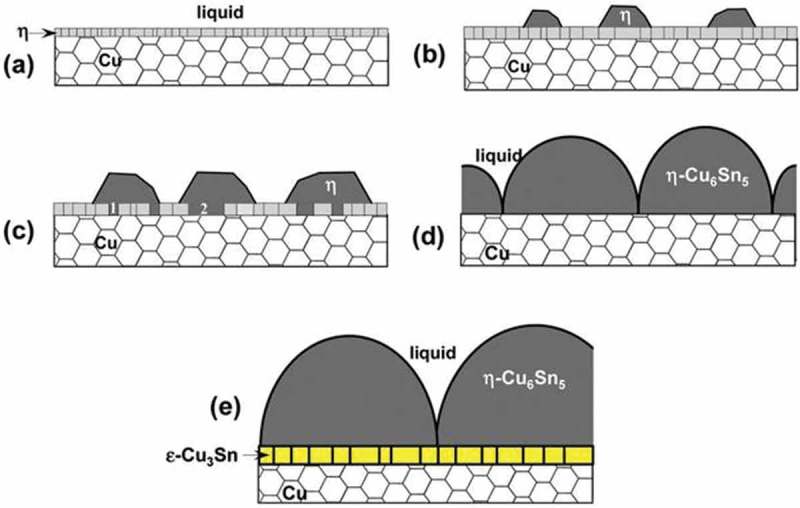
Schematic presentation of initial phase formation at Cu/liquid Sn interface. Reproduced with permission from [64].

It has been reported that the first phase of nucleation and growth at the Sn/Cu interface is Cu_6_Sn_5_. Under the condition that the thickness of the layer is less than several hundred nanometers, the growth of η-Cu_6_Sn_5_ will inhibit the nucleation and growth of the second phase ε-Cu_3_Sn [64]. Figure 7 shows the growth pattern of IMCs at the interface between Cu and liquid Sn.

- In the oversaturated Sn-Cu liquid alloys, the η-Cu_6_Sn_5_ phase nucleate heterogeneously at the liquid/Cu interface and grow continually until a successive thin layer of η-Cu_6_Sn_5_ with tens of nanometers thick is formed at the interface.
- The rapid diffusion of copper atoms passes through thin and continuous η-Cu_6_Sn_5_ boundaries, realizing the rapid growth of individual faceted grains (scallops) of η-Cu_6_Sn_5_ phase on a continuous thin layer.
- The scallop like Cu_6_Sn_5_ grains continue to grow at the interface along with the growth of ε-Cu_3_Sn phase at Cu/η-Cu_6_Sn_5_ interface.

The influence of Cu content on the interface IMC between Sn-Cu/Cu interface was researched [65]. It was found that the higher the soldering temperature, the thicker the Cu_6_Sn_5_ formed. At the same soldering temperature, with the increase of Cu content in the solder, the thickness of IMC layer was decreased and then increased. When the content of Cu is less than 1.1%, a part of Cu_6_Sn_5_ grains will dissolve into the solder, resulting in the decrease of IMC. While the content of Cu is higher than 1.1%, the Cu_6_Sn_5_ in the solder will deposit and the IMC layer will become thicker. El-Rehim and Zahran [66] investigated the effect of aging treatment at different temperatures on Sn-xCu (x = 1,2,3,4,5%) solder. Comparing the microstructure of Sn-1.0Cu aged at 393K and 433K, it was found that the growth of Cu_6_Sn_5_ was coarser at higher temperature. The Cu_6_Sn_5_ precipitated first during reflow and then the Cu_3_Sn precipitated after a certain time. The Cu_6_Sn_5_ and Cu_3_Sn both grow at the beginning of isothermal heat treatment. After consuming all available Sn, the Cu_3_Sn is formed at Cu/Cu_6_Sn_5_ interface at the expense of Cu and Cu_6_Sn_5_. Therefore, the increase of Cu content will make Cu_3_Sn more intensive.

**Figure 8.**
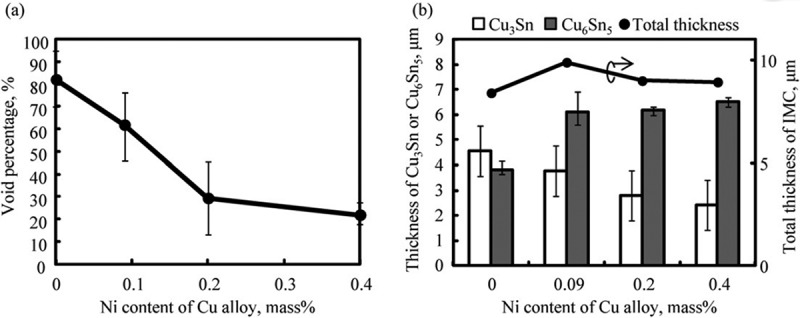
(a) Void percentage and (b) IMC layer thickness versus the Ni content in the Cu alloy following annealing at 423 K for 2000 h. Reproduced with permission from [71].

During the long time annealing, the crackle is formed at the interface between Cu substrate and Cu_3_Sn layer, leading to lower reliability of the solder joints. This influence is often caused by Kirkendall voids formed by different diffusion rates of Sn and Cu [67]. It has been confirmed that the addition of alloying elements can affect the growth behaviour of IMCs and the formation of Kirkendall voids [68,69]. Adding a small amount of Ni to Sn-0.7Cu alloy can significantly change the morphology of IMC, inhibit the formation of Cu_3_Sn layer and slow down the dissolution rate of the substrate [70]. Maeshima et al. [71] studied the effect of Ni addition to Cu substrate on the diffusion behavior of Cu and Sn atoms at the interface. Compared with Cu substrate without Ni addition, the addition of Ni effectively inhibited the formation of voids and the growth of Cu_3_Sn layer in the process of mutual diffusion between Cu substrate and Sn-0.7Cu solder. As shown in Figure 8, with the increase of Ni content, the void decreases, and the void rate decreases significantly when the content of Ni reaches 0.2%. In addition, with the increased of Ni content, the thickness of Cu_3_Sn layer decreased and the thickness of Cu_6_Sn_5_ layer increased. It was found that the void fraction was positively correlated with the Cu_3_Sn layer and negatively correlated with the Cu_6_Sn_5_ layer. Since the total IMC was composed of two compounds, the maximum thickness of IMC was obtained when the Ni content was 0.09%. The growth of Cu_3_Sn layer at the interface lies on the diffusion flux of Cu and Sn atoms in the substrate and the solder. The experimental results showed that the Ni addition did affect the diffusion flux of Cu and Sn atoms, thus affecting the growth rate of Cu_3_Sn.

The growth of Cu_6_Sn_5_ and Cu_3_Sn layer of Sn-0.7Cu solder containing TiO_2_ and Ni after isothermal annealing was investigated [72]. With the TiO_2_ addition, the scallop-like Cu_6_Sn_5_ phase became flatter and the interfacial boundary decreased. After a long time annealing, the Kirkendall voids appeared in the Cu_3_Sn layer, while in the solder joints with the Ni addition, the scallop-like (Cu,Ni)_6_Sn_5_ appeared to be finer, and the Cu_3_Sn was thinner, which indicated that Ni inhibited the diffusion of Cu from the substrate to the Cu_6_Sn_5_ layer, farther inhibiting the growth of Cu_3_Sn.

**Figure 9.**
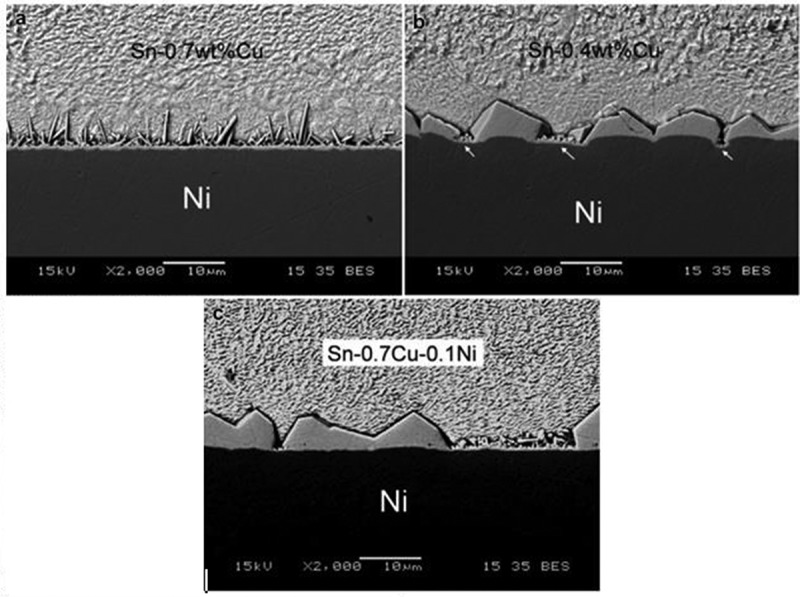
Backscattered electron images (BEIs) showing the interfaces between the solders and Ni at 250 °C for 3 min, (a) Sn-0.7Cu/Ni, (b) Sn-0.4Cu/Ni, and (c) Sn-0.7Cu-0.1Ni/Ni. Reproduced with permission from [77].

Ni element is also commonly used as a barrier layer between the Cu substrate and solder because it can slow down the metallurgical reaction rate during soldering [73,74]. The research of Sn-0.7Cu/Ni interfacial reaction showed that a thin layer of Ni_3_Sn_4_ will be formed at the Cu_6_Sn_5_/Ni interface with the continuous growth of Cu_6_Sn_5_ layer during soldering, and eventually Ni_3_Sn_4_ particles will diffuse into the solder melt. The evolution of this interfacial reaction is mainly influenced by the composition and grain size of solder, especially the Cu content in solder [75]. When the content of Cu increased to 0.4%, the interfacial reaction products changed from Ni_3_Sn_4_ to Cu_6_Sn_5_ and when the content of Cu was more than 0.6%, the layer of Cu_6_Sn_5_ appear peeling phenomenon [76]. The interface between Sn-0.7Cu and Sn-0.4Cu solder at 250 °C for 3 min is shown in Figure 9 [77]. The Cu_6_Sn_5_ phase of Sn-0.7Cu/Ni has a finer rodlike structure. In addition, the Ni substrate boundaries of Sn-0.7 Cu/Ni solder is straight and irregular for Sn-0.4Cu. As shown by the arrow in Figure 9(b), there are no more products at the concave interface, which reflects that the consumption rate of Ni here is faster, resulting in irregular shape of the substrate. As shown in Figure 9(c), the reaction phase morphology of Sn-0.7Cu-0.1Ni/Ni is similar to that of Sn-0.4Cu/Ni, but the interface is straight since the addition of Ni reduces the dissolution of Ni substrates. The nucleation sites of Cu_6_Sn_5_ increase with the increase of Cu content, and the nucleation mechanism is dominant. The morphology of Cu_6_Sn_5_ phase transforms into slender rod shape with the low solubility of Ni. In thermodynamics, if the Ni solubility is high, the Cu_6_Sn_5_ phase will be stabilized. Adding Ni to the solder contributes to the growth of Cu_6_Sn_5_ and hinders its nucleation. The growth mechanism plays a major role, resulting in a small number of large-sized Cu_6_Sn_5_ grains. Compared with Sn-0.7Cu/Ni, the interface in Sn-0.4Cu/Ni is obviously coarsened. Because of the low content of Cu, Ni dissolves into Sn-0.4Cu rapidly. At this time, the growth mechanism of Cu_6_Sn_5_ phase plays a major role, which also explains the coarsening of Cu_6_Sn_5_ phase in Sn-0.7Cu-0.1Ni/Ni.

On the other hand, the Ni addition may cause the spalling of solder joint at interface IMC layer, resulting in deterioration of solder joint performance [78,79]. Unlike the preferential substitution of Ni for the Cu atom in Cu_6_Sn_5_, the substitution of the Zn for Sn atom in the sublattice seems to lead to a more stable thermodynamic structure [80]. Zeng et al. [81] investigated the influence of aging on the phase stability of IMCs at the interface of Sn-Cu solder joints containing Ni, Zn and Au. It was found that Ni, Zn and Au inhibited the phase transformation of hexagonal to monoclinic Cu_6_Sn_5_ driven by aging, leading to the improvements in phase stability. This discovery helps to further comprehend the reliability of the interface structure of microalloyed lead-free solder joints.

The Al addition can cause significant changes in Sn-0.7Cu/Cu interface structure [69]. The interface IMC is Cu_6_Sn_5_ in Sn-0.7Cu-(0.01–0.025)Al solder and Al_2_Cu in Sn-0.7Cu-(0.05–0.075)Al solder, since the low solidification solubility of Al atoms in the matrix of β-Sn and the tendency to segregation in the interface during solidification, which makes the transition of Cu_6_Sn_5_ into δ-Al_2_Cu phase. The thickness of scallop-like IMC is mainly controlled by the change of IMC and the reaction between Al and Cu. The effect of (0.05–0.075)Al addition on the thickness of IMC layer is weak. But the thin brittle interfacial IMC is conducive to the bonding strength and reliability of the solder joints [82]. A small amount of Al addition to Sn-0.5Cu solder alloy can contribute significant change of the microstructure too [83]. The Cu-Al IMC with rhombic hexahedral structure was determined by transmission electron microscopy and electron probe microanalysis. The addition of Al in the soldering alloy was beneficial to the formation of Cu-Al IMC in the soldering alloy matrix, named Cu_33_Al_17_, which resulted in the refinement of Cu_6_Sn_5_ in the solder alloy. The solder alloy with 0.03%Al exhibited the best properties.

**Figure 10.**
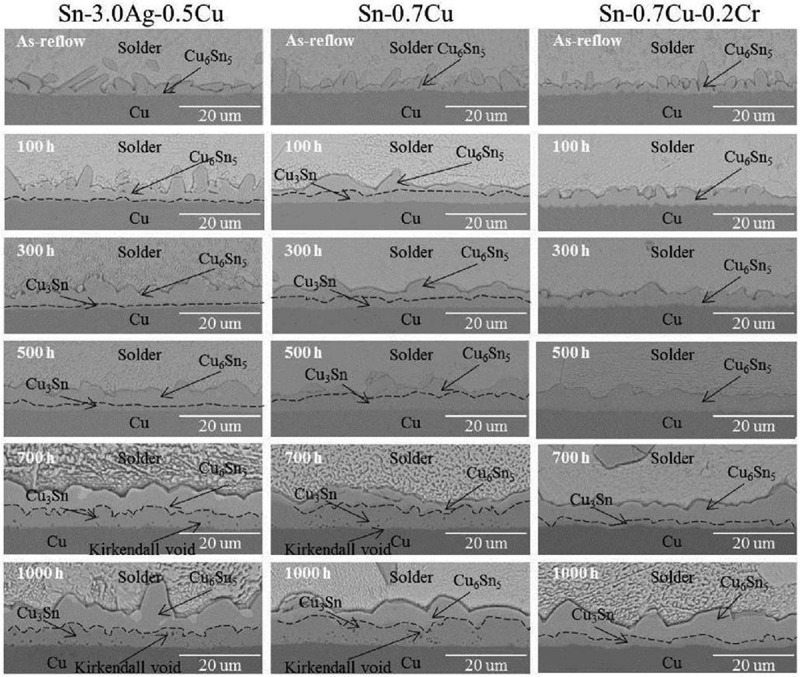
Cross-sectional SEM images of IMC layers after aging at 150 °C for up to 1000 h. Reproduced with permission from [87].

The influence of Sb addition on the interface reaction of Sn-0.7Cu/Cu was investigated [84]. When the content reached 0.5%, the IMC morphology of solder joint interface changed greatly, the scallop IMC was refined and the thickness of IMC layer was the smallest under this condition. As the content increased, the IMC grains coarsened and the thickness of the interface layer increased gradually. It is reported that the addition of microscale Cr can effectively inhibit the growth of Cu-Sn IMC [85,86]. Figure 10 shows that for Sn-3.0Ag-0.5Cu and Sn-0.7Cu solders, the thin Cu_3_Sn IMC layers are formed at the Cu_6_Sn_5_/Cu interface after aging at 150 °C for 100h [87]. A similar phenomenon occurs only when the Sn-0.7Cu-0.2Cr solder alloy is aged at 150 °C for 500h and the thickness of IMC containing Cr seems to be thinnest at any stage. In thermodynamics, the formation of Cu_3_Sn IMCs between Cu_6_Sn_5_ IMC layer and Cu is spontaneous, but this can be dominated by kinetic parameters, such as the diffusion of Sn to Cu_6_Sn_5_ IMCs or Cu substrate. The authors insisted that Cr inhibited the growth of IMC at solid-state interface by slowing down the diffusion rate of Cu. Unlike Ni atom, the Cr atom can not form the solid solution in CuSn IMC. Many CrSn_2_ particles take root at the interface and undergo mature growth during aging. The inhibition behavior may be related to the existence of small particles at the interface and the influence of the flow of Cr and Sn atoms on the diffusion of Cu and the bonding of Sn and Cu atoms.

**Figure 11.**
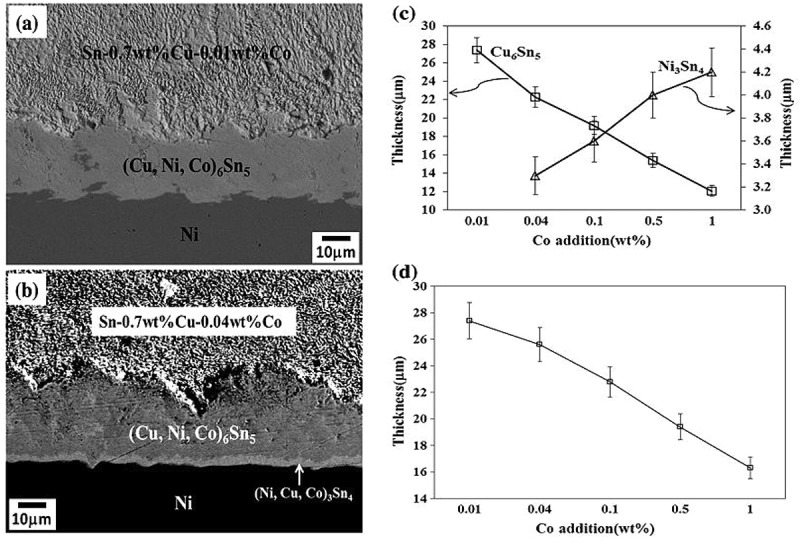
(a) BEI of the Sn-0.7Cu-0.01Co/Ni interfacial reaction at 250 °C for 30 min. (b) BEI micrograph of the Sn-0.7Cu-0.04Co/Ni interfacial reaction at 250 °C for 30 min. (c) Thickness of the Cu_6_Sn_5_ and Ni_3_Sn_4_ phases in Sn-0.7Cu-Co/Ni couples reacted at 250 °C for 30 min. (d) Total thickness of theCu_6_Sn_5_ and Ni_3_Sn_4_ phases in Sn-0.7Cu-Co/Ni couples reacted at 250 °C for 30 min. Reproduced with permission from [93].

Among the alloying elements researched, Co is also potential based on its positive influences [88,89]. The effects of minor Co additions on interface reactions of solders/Cu substrate have been investigated in the literature [90–92]. The micrographs of Sn-0.7Cu-Co/Ni interfacial reactions with Co additions were shown in Figure 11(a,b) [93]. Compared to the 0.01%Co addition, when the amount of Co addition increased to 0.04%, Ni_3_Sn_4_ phase beneath the Cu_6_Sn_5_ phase can be observed. Along with Co additions, the thicknesses of the Cu_6_Sn_5_ and Ni_3_Sn_4_ phases decreased and increased, respectively, and the total thicknesses decreased.

**Figure 12.**
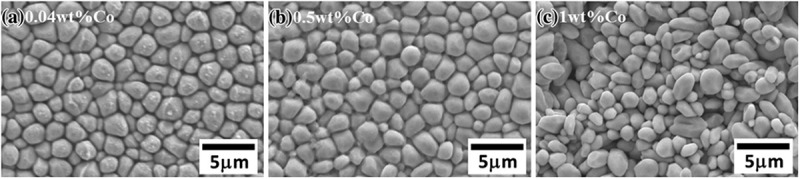
Grain morphology of the (Cu, Co)_6_Sn_5_ phase formed in Sn-0.7Cu-Co/Cu couples. Reproduced with permission from [93].

**Figure 13.**
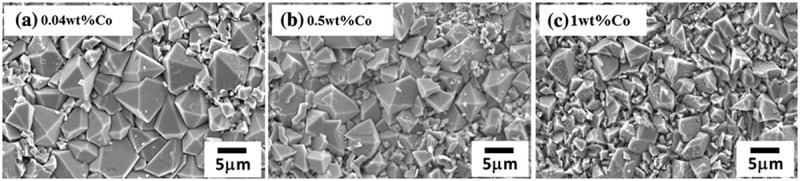
Grain morphology of the (Cu, Ni, Co)_6_Sn_5_ phase formed in Sn-0.7Cu-Co/Ni couples. Reproduced with permission from [93].

Remarkably, the dissolution of Co in Cu_6_Sn_5_ and (Cu, Ni)_6_Sn_5_ phases has no effect on the grain morphology but it does refine the grains on either substrate, as shown in Figures 12 and 13 [93]. With Co additions, the more sites for Cu_6_Sn_5_ phase nucleation were provided, because the catalysing effect of Co, hence more Cu_6_Sn_5_ was refined.

**Figure 14.**
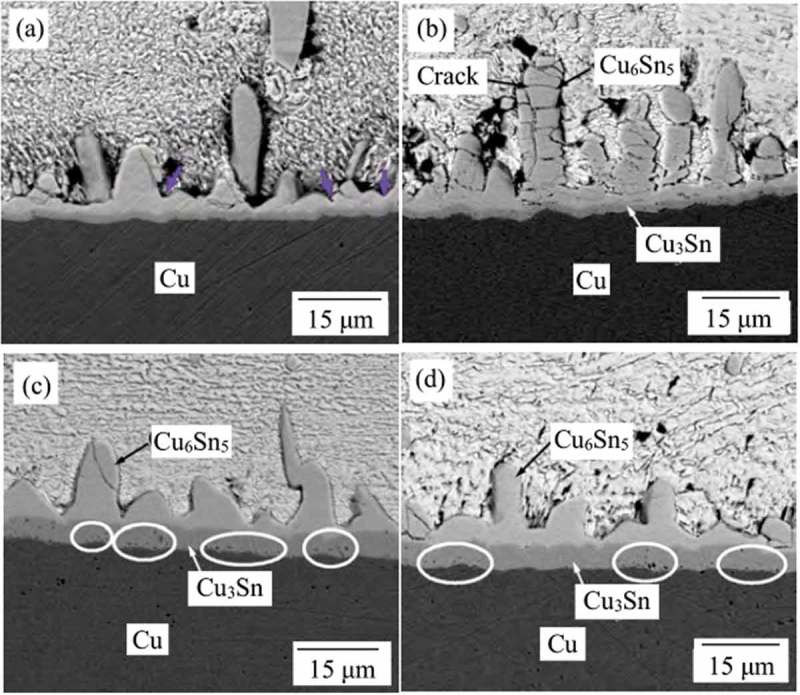
Cross-sectional SEM images of Sn0.7Cu0.5Er/Cu interfaces with isothermal aging (a) 1d; (b) 5d; (c) 7d; (d) 9d. Reproduced with permission from [95].

The influencing mechanism of rare earth elements is also a hot topic in the research of lead-free interconnects. The trace amount of Ce (about 0.1%) could inhibit the growth of IMC layer in Sn-Cu solder joints, but adding excessive Ce could promote the growth of IMC layer, which may be caused by the different effects of different amounts of Ce on the diffusion behaviour of Cu at the interface [94]. In addition, with the increase of temperature, the effect of Ce on interface reaction weakened gradually. Hu et al. [95] investigated the interface reaction of Sn-0.7Cu-xEr/Cu solder joints. It was found that the rare earth element Er inhibited the growth of IMC layer (including Cu_6_Sn_5_ and Cu_3_Sn), and the higher the content of Er within tolerable limits, the stronger the inhibition. It depends not only on the concentration gradient of elements but also on the activity of elements. Trace rare earth Er is insoluble in the Sn matrix but has the Sn-affinity to react with Sn, which reduces the activity of Sn and further inhibits the growth of Cu_6_Sn_5_ IMC layer. As shown in Figure 14, after isothermal aging, the grain size of IMC layer grows obviously, the morphology of Cu_6_Sn_5_ phase becomes flat, the Cu_3_Sn layer thickens sharply and some Kirkendall voids appear at the interface of Cu_3_Sn/Cu. Remarkably, the voids in the joints with 0.5% Er are less than those with 0.1% Er.

**Figure 15.**
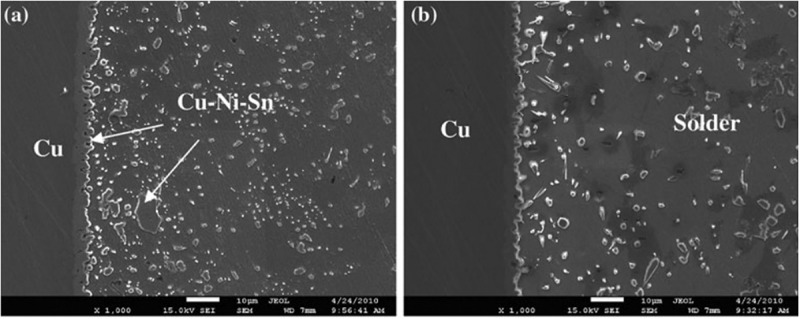
SEM cross-sectional micrograph of the as-soldered Sn-Cu-Ni-xPr joint. (a) x = 0, (b) x = 0.05wt%. Reproduced with permission from [53].

The addition of rare earth Pr and Nd can effectively change the morphology of IMC layer in the interface reaction of Sn-0.7Cu-0.05Ni-xPr(Nd)/Cu solder joints [53,54]. As shown in Figure 15, with the addition of 0.05% Pr, the thickness of IMC layer decreases significantly and the formation of voids at the interface is inhibited. Because of the good affinity between Pr and Sn, the two elements are easy to form stable compounds at the interface and prevent the reaction of Sn and other elements to some extent, reducing the activity of Sn at the interface, thus inhibiting the interface reaction of solder/Cu substrate. On the other hand, PrSn_3_/NdSn_3_ formed by Pr/Nd reacting with Sn has the higher melting point, so it will precipitate preferentially during solidification, forming heterogeneous nucleation particles, promoting solidification of solder alloys, shortening the interface reaction time and inhibiting the growth of IMCs.

A part of the minor elements effects on the IMCs based on the current research data of Sn-Cu lead-free solders are provided in Table 2.

**Table 2.** Effects of elements on the interfacial IMCs.

Elements/compounds	Cu_6_Sn_5_ growth	Cu_3_Sn growth	Thickness of IMC	Ref.
Ni	Decrease	–	Decrease	[19]
Ni	–	Decrease	Decrease	[53]
Ni	Slight increase	Decrease	No influence	[71]
Al	Decrease	–	Decrease	[40]
Ag	Increase	–	Increase	[42]
Zn	Slight increase	–	Slight increase	[45]
Zn	Slight decrease	Slight decrease	Slight decrease	[46]
Pr	Slight decrease	Slight decrease	Slight decrease	[53]
TiO_2_	Decrease	Slight increase	Slight decrease	[72]
Sb (<0.5wt.%)	Decrease	–	Decrease	[84]
Cr	Decrease	Decrease	Decrease	[87]

The phase field method has been used for the simulation of interfacial reactions. The effects of grain boundary diffusion coefficient and interfacial energy between primary IMC and liquid Sn on the microstructure evolution and growth kinetics of Cu_6_Sn_5_ interface in Sn/Cu interconnection system were researched by using the multiphase field model [96]. It was found that the thickness of IMC was positively correlated with the grain boundary diffusion coefficient and negatively correlated with the interfacial energy, whereas the average transverse grain size of Cu_6_Sn_5_ was inversely correlated with them. Combining the 2D and 3D simulation results of IMC morphology evolution at the interface with the experimental results, the simulation results showed good agreement with the experimental results. Ma [97] used a binary alloy phase field model to simulate the formation, morphological evolution and growth of Kirkendall voids in the Cu/Cu_3_Sn interface and IMC layer of Sn/Cu interconnection system. The effects of interfacial Cu_3_Sn layer thickness and impurity content on the morphology and growth kinetics of Kirkendall voids were simulated and analysed. The results showed that a large number of atom mismatch regions can be formed at the Cu/Cu_3_Sn interface, and then grew into Kirkendall voids. With the aging process, the voids grew and merged. In addition, both the number and size of Kirkendall voids were positively correlated with Cu_3_Sn layer thickness and impurity content.

## 4. Melting characteristics

The basic thermal properties of eutectic Sn-0.7Cu solder alloy are shown in Table 3 [98]. The melting characteristics of lead-free solders are similar to those of Sn-Pb solders. That is beneficial to follow the existing soldering process and equipment. On the other hand, it is vital to reduce the damage of circuit board components caused by heat output during brazing as far as possible.

**Table 3.** Basic thermal properties of eutectic Sn-0.7Cu solder alloy [98].

Property	Value
Eutectic temperature (°C)	227
Latent heat of fusion (kJ/kg)	44.37
Thermal conductivity (W/m·K)	53
Specific heat (J/kg-K)	223
Density (kg/m^3^)	7300

Usually, the element doping can affect the melting characteristics of Sn-Cu binary alloy, but the effect seems to be rather weak. For example, the addition of Bi and P can reduce the melting point of Sn-0.7Cu alloy in a small range [99]. After adding 2% Ag and 2% In to the Sn-0.7Cu solder alloy [43], the melting point decreased from 227.4 °C to 224.0 °C and 217.8 °C, respectively, which could be attributable to softening and dissolution of second phase particles. In other words, with Ag and In addition, the formation of reinforcing IMC phase leads to the increase of surface instability and the transformation in physical properties of the grain boundary/interfacial characteristics. Li et al. [100] considered that the melting point was not significantly affected when the content of In in Sn-0.7Cu-0.2Ni solder was less than 0.3%, but the melting range of the solder increased obviously.

The addition of Co in Sn-0.7Cu alloy advances the formation of (Cu,Co)_6_Sn_5_ and (Cu,Co)Sn_2_ IMCs which seem to play a role in stabilizing microstructure [101], while with the increase of Co content, the melting point of composite solder alloy tends to increase (Sn-0.7Cu-2Co alloy melting point is 232.2 °C) [102]. The purpose of adding these components is to maximize the mechanical and other properties of the solder alloy and improve the reliability of lead-free solder joints on the premise of not excessively increasing the melting point of the alloy. This is a worthwhile idea for the modification of lead-free solder. Table 4 shows the peak temperatures for each vertex based on the differential scanning calorimetry (DSC) data of three solder alloys. The addition of Ti can effectively reduce the undercooling of solder, thus changing the solidification behavior and microstructures of solder alloys [103].

**Table 4.** Summary of peak temperatures (°C) and undercooling (ΔT) in DSC analysis for Sn–Cu base solders [103].

	Heating process	Cooling process	Undercooling
	*T*_p_ (eutectic&β-Sn)	*T*_p_ (eutectic&β-Sn)	Δ*T* (β-Sn)
Sn-0.7Cu	229.4	194.9	34.5
Sn-0.7Cu-0.2Ti	230.2	226.3	3.9
Sn-0.7Cu-0.6Ti	230.2	226.6	3.6

Adding TiO_2_ nano-particles to Sn-0.7Cu solder alloy can slightly improve the melting properties. With the increase of the content of TiO_2_, the solidus and liquidus temperatures and melting range of the alloy decrease slightly, which may be due to the increase of the surface-free energy of the solder alloy caused by the TiO_2_ particles addition. Moreover, the properties of TiO_2_/interface at the grain boundaries of the solder were changed significantly, which resulted in the change of the physical properties [50]. Table 5 provides a summary of the alloy composition and the data of melting points based on the current research of Sn-Cu lead-free solder.

**Table 5.** Melting points of selected Sn-Cu solder alloys.

Solder alloy	T_m_ (°C)	Ref.
Sn-0.7Cu	228	[87]
Sn-0.7Cu-0.4Co	224	[101]
Sn-3Ag-0.5Cu	219	[87]
Sn-0.7Cu-0.2Cr	231	[87]
Sn-0.7Cu-0.25TiO_2_	226–229	[50]
Sn-4Cu	360–368	[38]
Sn-4Cu-0.02Al	340–346	[38]
Sn-4Cu-0.2Al	317–321	[38]
Sn-0.7Cu-0.2Ni	226–229	[100]
Sn-0.7Cu-0.2Ni-0.1In	226–230	[100]
Sn-0.7Cu-0.2Ni-0.2In	226–230	[100]
Sn-0.7Cu-0.2Ni-0.3In	225–229	[100]

A large number of researches have shown that the effect of the element addition on the melting point of Sn-Cu solder alloy seems not to be remarkable, but the refinement of solder microstructure and the strengthening of other properties by element doping could not be neglected. To solve the problem of excessive melting point of Sn-Cu solder, the research can start with the solder alloy itself or the melting range of the solder which could be shorten by optimizing the soldering parameters, such as the rapid solidification technology [104,105] and directional solidification technology [106]. The spreading and melting of the solder can be realized in a short time, reducing the heat release and the thermal damage of the electronic components during the soldering process. In addition, it has been reported that the atomization and extrusion of solder alloy can improve the melting properties and other mechanical properties of solder alloy [107].

## 5. Wettability

Wettability refers to the ability of a liquid metal to wet and spread on the parent metal surface. It can also be described as the trend of liquid spreading on solid substrates [108]. Wettability is determined by the surface and interfacial energy involved in the solid/liquid interface, which is influenced by many factors, such as liquid viscosity, interfacial reaction and thermal conditions of the system [109]. Several important indicators of wettability evaluation include the contact angle, wetting force, wetting time. The wetting and spreading of solder on the surface of solid base metal are mainly caused by the different interfacial tension between solid and liquid and the surface tension of liquid. After the spreading and wetting of droplets on the solid plane interface, it will eventually reach equilibrium state, that is, the surface tension at the three-phase boundary points of the system will reach equilibrium. This state can be described by Young’s equation:

(1) $$\sigma_{SG}=\sigma_{SL}+\sigma_{LG}\cos\theta$$

where σ_SG_ is the surface tension acting on droplets along boundary between solid and gas, σ_SL_ the surface stress on solid-liquid boundary, σ_LG_ the surface tension of liquid on the boundary of gas medium and *θ* the wetting angle in equilibrium state.

In the actual soldering process, there will be violent interaction between base metal and solder. In this case, the surface wetting, spreading and capillary phenomena are more complex, so the above equation is only an approximate description.

Table 6 describes the natural radius of curvature, *R*, of the alloys, calculated by Equation (2). Compared with Sn-40Pb, Sn-0.7Cu solder alloy has a larger natural curvature radius. The implication of this result is that the phenomenon of bridging must be given special attention, along with the trend of decreasing solder joint spacing.

(2) $$R=\gamma \rho g^{1/2}$$

**Table 6.** Natural radius of curvature R, of solder alloys [24].

Alloy	*R*_air_ (mm)	*R*_nitrogen_ (mm)
Sn-40Pb	2.19	2.31
Sn-0.7Cu	2.62	2.54

where *γ* is the surface tension, *ρ* the density and *g* the acceleration due to gravity.

During soldering, the viscosity of solder alloy directly affects gap filling and weldability. The viscosity of Sn-0.7Cu solder alloy decreases with the increase of temperature [110]. But with the increase of temperature, the liquid structure of the melt could be changed, and the surface tension of the solder first decreases to a certain value and then increases slightly. If only the wettability of solder is considered, the optimum soldering temperature for Sn-0.7Cu alloy is 303 °C, under which the minimum surface tension is about 369 mN/m [22]. The surface tension of Sn-based binary lead-free solder alloy can be calculated and its wettability can be evaluated by melt viscosity value based on the expression of surface tension and viscosity [111]. The relationship between surface tension and viscosity in monolithic system and vacuum is deduced as follows:

(3) $$\frac{\gamma }{\eta }=\frac{15}{16}\sqrt{\frac{kT}{m}}$$

Where *γ* is the surface tension, *η* is the viscosity, *k* is the Boltzmann constant, *T* is the absolute temperature, and *m* is the atomic mass. In addition, the viscosity of the melt is approximately expressed by the following equation [112]:

(4) $$\eta =hv_{m}exp\varepsilon kT$$

where h is Planck’s constant, v_m_ is the flow unit volume and ε is the activation energy to move the flow unit in the melt.

**Figure 16.**
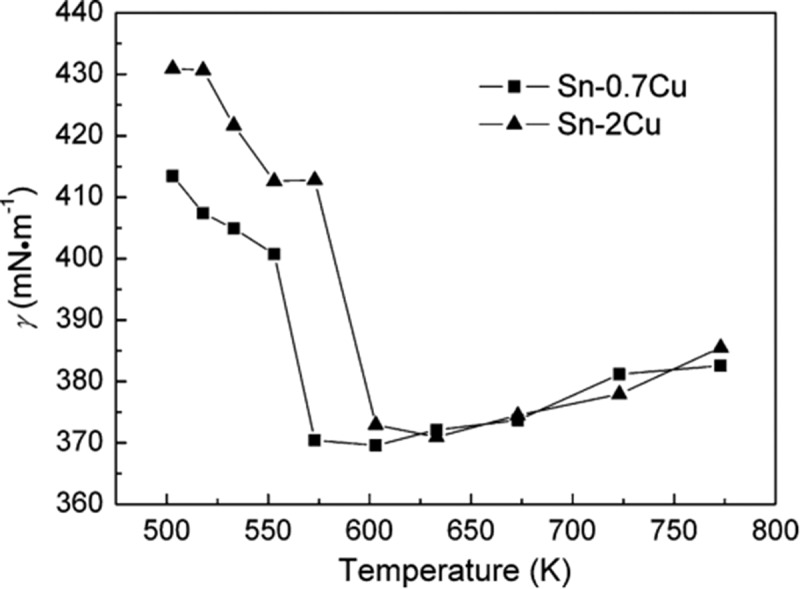
Surface tension of liquid Sn-Cu solders. Reproduced with permission from [110].

As shown in Figure 16 [110], the surface tension of liquid Sn-Cu solders at different temperatures was calculated, based on the above two formulas. The surface tension of the two kinds of solder is similar. The smallest values were obtained at 633 K for Sn-2Cu and 603 K for Sn-0.7Cu, respectively.

**Figure 17.**
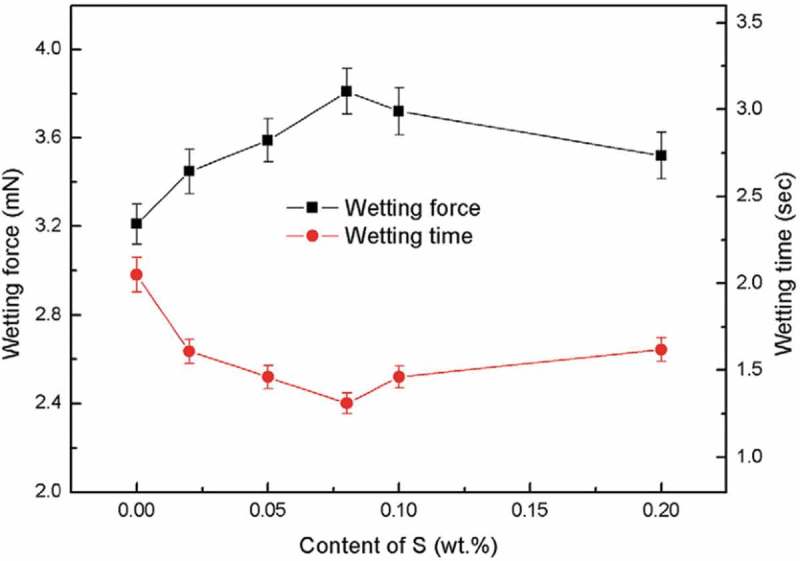
Wettability of Sn-0.7Cu-xS on Cu sheet in wetting balance tests. Reproduced with permission from [115].

It is a practical and effective method to study the influence of element doping on the wettability of solder under laboratory conditions. Huang et al. [113] researched the effect of S element on the wettability of Sn-0.7Cu solder on Cu substrate in 3.5% NaCl solution. Figure 17 shows that the wetting performance of Sn-0.7Cu solder can be greatly improved by adding 0.08% S. S is easy to concentrate on the surface of the solder melt during the soldering process, which reduces the surface tension of the alloy since the non-metallic element S is difficult to dissolve into the alloy. In addition, the addition of S inhibits the oxidation of Sn. These two mechanisms promote the spreading of solder melt on the Cu substrate. On the other hand, the formation of SnS and SnSO_4_ by the addition of excess S hinders the flow of solder melt and cancels the positive effect.

**Figure 18.**
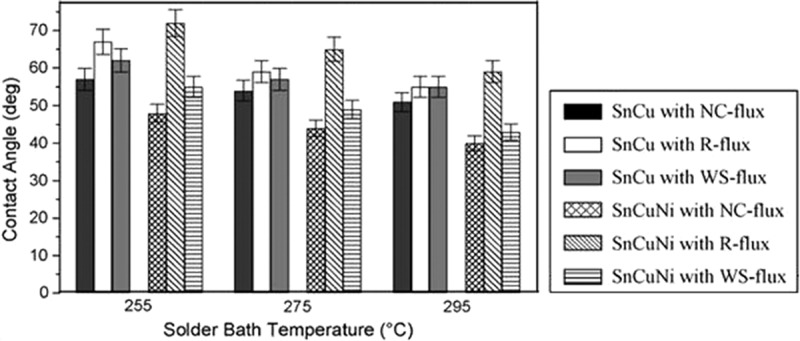
Comparative contact angle formations with Cu-substrate. Reproduced with permission from [111].

**Figure 19.**
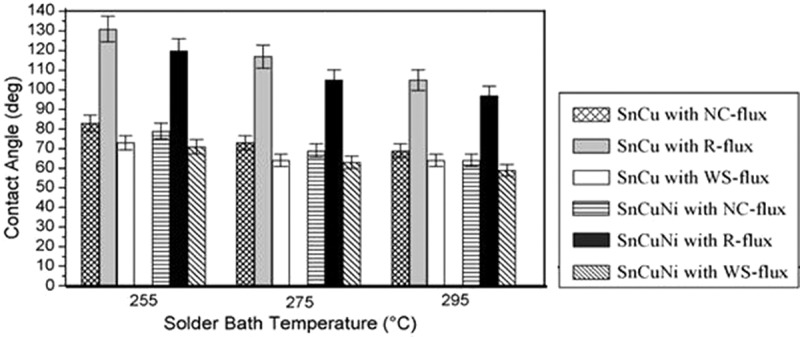
Comparative contact angle formations with Ni-substrate. Reproduced with permission from [111].

The effect of Ni content on the wettability of Sn-Cu-xNi solder on Cu substrate was investigated by Silva et al. [114]. It was found that Sn-0.7Cu solder with 0.05%Ni had smaller wetting angle, and the relationship between initial contact angle and heat transfer coefficient was revealed. In addition, with 0.3% In addition to the Sn-0.7Cu-0.2Ni soldering alloy, the optimum thermal expansion coefficient of the soldering alloy was 17.5 × 10^−6^/°C. The spreading area of the soldering alloy increase by 15.6% with the increase of thermal expansion coefficient [99]. The contact angles of Sn-0.7Cu-0.3Ni and Sn-0.7Cu solder under different conditions are shown in Figures 18 and 19 [111]. The lower contact angles with Cu substrate for both solder is found by using no-clean (NC) flux. Analogously, the lower contact angles with Ni substrate for both solder is found by using water-soluble (WS) flux. It seems that the non-activated (R) flux is not suitable for both solder, especially on Ni substrate. In addition, with the rise of solder bath temperatures, both the contact angle of Sn-0.7Cu and Sn-0.7Cu-0.3Ni solder decreases. Moreover, with the addition of 0.3Ni into the Sn-Cu solder shows better wettability.

Zhu et al. [45] researched the influence of Zn particles on the wettability of Sn-0.7Cu solder. With the increase of Zn particles, the spreading coefficient of the solder decreased first and then increased. With the addition of 0.2% Zn particles, the spreading coefficient was the largest (79.43%). When the addition is more than 0.2%, the spreading coefficient of the solder decreased, which is due to the addition of trace Zn particles increase the effective amount of heterogeneous nucleation on the substrate and promote the growth of interfacial IMC. The appropriate thickness of interfacial IMC layer is helpful to the flow of the solder melt on the Cu substrate, thus improving the spreading performance. However, the excessive Zn particles could produce agglomeration phenomenon and float on the surface of the solder melt, which increased the viscosity of the solder melt and reduces its fluidity, thus showing a poorer spreading performance.

**Figure 20.**
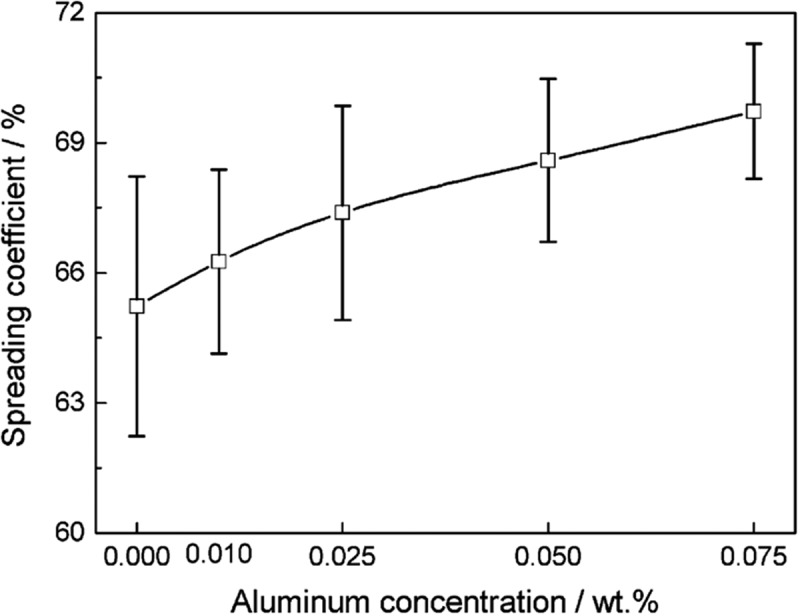
Spreading coefficient of Sn-0.7Cu-xAl (x = 0 ~ 0.075) lead-free solder alloys. Reproduced with permission from [39].

With the increase of Al concentration, the spreading coefficient of Sn-Cu-Al solder increases. Figure 20 shows the relationship between Al content and the diffusion coefficient of Sn-Cu-Al solder alloy on the Cu substrate [39]. When the Al content reaches 0.075%, the diffusion coefficient of the solder alloy increases to about 70% from the initial value of 65.23%. It is evident that the addition of Al promotes the wetting of solder alloys, which attributes to the decrease of surface tension of liquid solders due to the high activity of Al element. In addition, the metallurgical reaction of forming Al_2_Cu phase consumes part of the Cu from the substrate, thus promoting the spread of solder [40].

**Figure 21.**
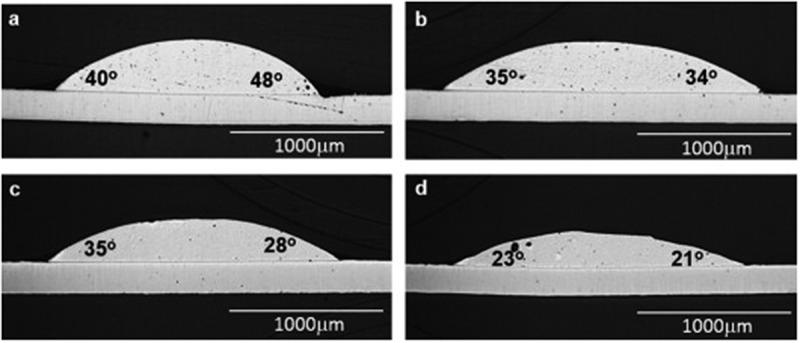
The wettability of solder alloys after reflow on Cu substrate at 250 °C for 90 s: cross-sectional images of (a) Sn-0.7Cu, (b) Sn-0.7Cu-0.01Pd, (c) Sn-0.7Cu-0.01Pd-0.15Cr and (d) Sn-0.7Cu-0.01Pd-0.15Cr-0.1Ca. Reproduced with permission from [85].

The effects of Cr, Ca and Pd on the wettability of Sn-Cu/Cu were investigated [85]. Typical contact angles of several reflowed samples on Cu substrates are shown in Figure 21. The results of the wetting angles show that Cr and Ca enhance the wettability of Sn0.7Cu solder alloy. Similar to Al element, the addition of Cr element can produce many thin rod-like CrSn_2_ IMC at the interface, resulting in lower surface tension. While the Ca addition can refine eutectic phase at the grain boundary, making rod-like IMC thinner near the interface, which is conducive to the spread of solder melt on the substrate.

It is noted that the wetting behaviour of Sn-Cu lead-free solder is affected significantly by the substrate material, temperature and flux. As shown in Table 7, the wetting behaviour of Sn-Cu lead-free solders by a part of the alloying elements reported is summarized.

**Table 7.** Results of wettability test from the literature.

Solder	Substrate	Flux	Temperature (°C)	Wetting force (mN)	Contact angle (°)	Ref.
Sn-0.7Cu	Cu	*NC*	255/275/295	1.39/1.54/1.65	57/54/52	[111]
Sn-0.7Cu-0.05Ni-0.05Pr	Cu	*NC*	255/275/295	2.85/3.2/3.55	–	[53]
Sn-0.7Cu	Cu	*WS*	255/275/295	1.33/1.58/1.71	62/58/54	[111]
Sn-0.7Cu	Cu	*R*	255	0.92	67	[111]
Sn-0.7Cu-0.3Ni	Cu	*R*	255	1.12	72	[111]
Sn-0.7Cu-0.3Ni	Cu	*NC*	255/275/295	1.69/1.94/2.05	47/44/41	[111]
Sn-0.7Cu-0.3Ni	Cu	*WS*	255/275/295	1.48/1.75/1.96	55/49/43	[111]
Sn-0.7Cu	Ni	*NC*	255/275/295	0.19/0.46/0.56	83/75/70	[111]
Sn-0.7Cu	Ni	*WS*	255/275	0.51/0.72	73/66	[111]
Sn-0.7Cu-0.3Ni	Ni	*NC*	255/275/295	0.29/0.56/0.68	79/72/68	[111]
Sn-0.7Cu-0.3Ni	Ni	*WS*	255/275	0.57/0.79	72/66	[111]
Sn-0.5Cu-0.05Ni	Cu	*NC*	240/260/280	1.59/3.41/3.62	–	[8]
Sn-0.5Cu-0.05Ni	Cu	*NC*/N_2_	240/260/280	3.23/3.62/3.78	–	[8]
Sn-0.5Cu-0.05Ni-0.05Ce	Cu	*NC*	240/260/280	2.89/3.55/3.90	–	[8]
Sn-0.5Cu-0.05Ni-0.05Ce	Cu	*NC*/N_2_	240/260/280	3.62/3.79/3.94	–	[8]
Sn-0.7Cu	Cu	*R*	260	3.21	39	[115]
Sn-0.7Cu-0.08S	Cu	*R*	260	3.80	32	[115]

*NC* no clean, *R* non-activated, *WS* water-solute

A large number of studies have shown that the wetting in the actual soldering process is related to the properties of the solder, but mainly depends on the role of the soldering fluxes [115–117]. Moreover, the influence of different kinds of flux on the wettability of solder is significant, for example, the differences in contact angles for Sn-0.7Cu and Sn-0.7Cu-0.3Ni solders by three different fluxes [111]. Under atmospheric conditions, the surface of the solder metal and substrate metal will be covered with a layer of the oxide film, which is very unfavourable to the spread of the solder melt on the substrate metal. In the soldering process, the use of fluxes can remove the oxides on the surface of the solder and substrate metal well, creating good conditions for the solder to infiltrate in the substrate metal. In addition, the flux can cover the substrate metal and the solder surface with a thin liquid layer to avoid the occurrence of secondary oxidation. Finally, it can enhance the fluidity and heat transfer capacity of the solder melt due to the activity of the flux. The fluxes should satisfy the following requirements as far as possible: (a) Dissolve or destroy the oxide film on the solder and substrate metal; (b) Suitable range of activity temperature; (c) Good thermal stability; (d) Small viscosity and density, good fluidity; (e) No malignant corrosion of solder joints; (f) Easy removal of post soldering residue; (g) Reasonable economy.

## 6. Mechanical properties and creep

The mechanical behaviour of solder is vital to the strength of the solder joints. Although the solder volume, joint geometry, bonding process and solidification rate have different effects on mechanical properties, the most essential factor is the alloy composition of solder. The effects of different element doping on the mechanical properties of Sn-Cu solder alloys were discussed, including tensile shear properties, hardness values and creep properties.

The ultimate tensile strength (UTS) of Sn-0.7Cu solder alloy is about 30 MPa at room temperature [52]. Hung et al. [31] found that when the Cu content of Sn-xCu solder increased, the Cu-Sn IMC increased and the Sn-rich phase refined. For the samples with low Cu content (0.3Cu and 0.6Cu), they have the good mechanical properties and long fatigue life due to the layered deformation structure of Sn-rich phase. The brittle and hard Cu_6_Sn_5_ phase can reduce the mechanical properties of solder joints, but when the Cu content reaches more than 1.3%, the grains are refined and the Cu_6_Sn_5_ phases are dispersed which must be one reason that the deformation resistance of Sn-rich phase strengthened and the stress-centralized effect of the Cu_6_Sn_5_ phase decreased.

**Figure 22.**
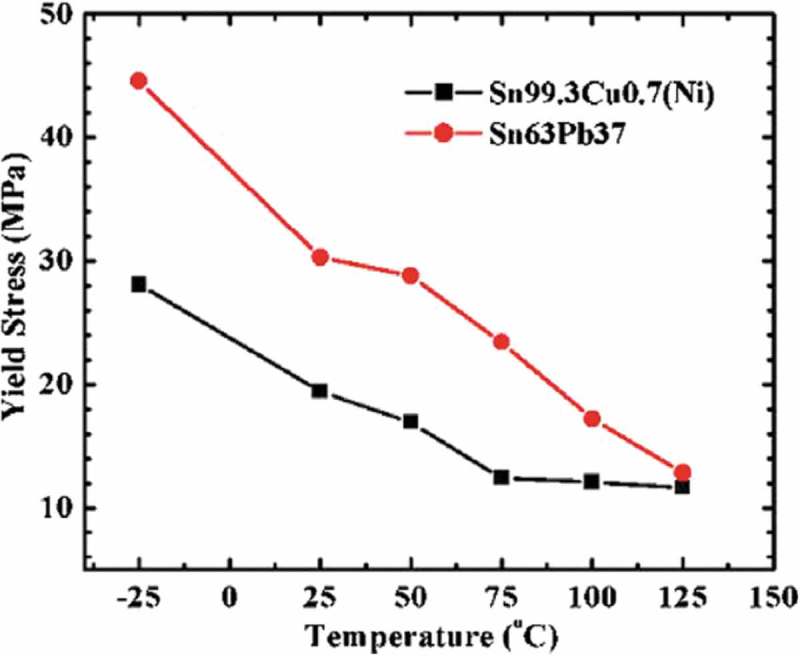
The ultimate tensile strength of two solder alloys Sn37Pb and Sn0.7Cu(Ni) in the temperature range from −25 °C to 125 °C (with a constant strain rate of 1.17 × 10^−3^ S^−1^). Reproduced with permission from [118].

The mechanical properties of Sn-0.7Cu(Ni) solder alloy exhibit strong temperature dependence and strain rate sensitivity in tensile tests at different temperatures and strain rates [118]. Figure 22 shows that yield strength (YS) of Sn0.7Cu(Ni) is smaller than that of Sn-37Pb, and both decrease with the increase of temperature. Alam et al. [57] added different amounts of nano-Cu particles in Sn by microwave sintering assisted powder metallurgy route to obtain compact materials with nearly equiaxed grains. Tensile test results show that, compared with Sn-0.7%Cu solder, adding 0.35vol.% (0.43wt.%) Cu in pure Sn can significantly improve the YS of solder alloys to 233% and UTS to 159%. The main feature of this preparation method is its cost-effectiveness.

The mechanical properties of the Sn-Cu lead-free solder alloy were improved, similar to the traditional Sn-Pb solder, by adding the trace alloying elements to the alloy. The mechanical properties of Sn-Cu solder are significantly affected by the addition of Al [39,40]. The UTS of the Sn-Cu-(0.01 ~ 0.025)Al solder alloy was deteriorated by the coarsened Cu_6_Sn_5_/Sn interface. The microstructure of Sn-Cu solder is refined by micro Al addition, but the tensile strength of Sn-Cu decreases with the increase of volume fraction of the brittle phase. With the increase of Al content (0.05 ~ 0.075), the formation of Cu_6_Sn_5_ IMC was inhibited. The precipitated fine Al_2_Cu acted as strengthening phase, which hindered the movement of tensile dislocation, and was beneficial to the improvement of UTS of solder joint. On the other hand, the UTS of Sn-Cu-Al solder is related to the distribution and strength of precipitated phases. The microhardness values of Al_2_Cu/Sn eutectic and β-Sn are 76.3 MPa and 11.7 MPa, respectively. The improvement of UTS of Sn-Cu-Al solder alloy is the result of the refinement structure of Sn-Cu-0.075Al alloy and the interaction of Al_2_Cu/Sn eutectic with high microhardness. Hasnine M and Vahora N [119] added 0.01% Ge to Sn-0.7Cu solder alloy. The results showed that the microhardness of as-cast alloy increased by 6.7%, which was attributed to the refinement of grain structure and the uniform distribution of IMC in eutectic phase caused by the addition of Ge.

**Figure 23.**
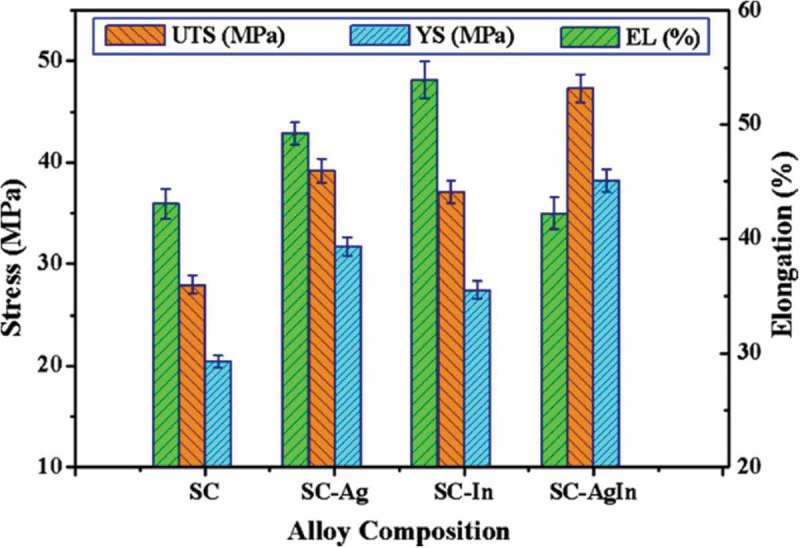
Mechanical properties of the samples. UTS: ultimate tensile strength; YS: yield strength; EL: total elongation; SC: Sn0.7Cu. Reproduced with permission from [44].

The experimental results of mechanical tests of Sn-Cu solder alloy with Ag and In addition is shown in Figure 23 [44]. The Sn-0.7Cu-2Ag-2In solder alloys exhibit the highest tensile strength 47.3 MPa, which is approximately 1.7, 1.3 and 1.2 times that of Sn-0.7Cu, Sn-0.7Cu-2In and Sn-0.7Cu-2Ag alloys, respectively. Also, for the same strain rate, the YS of Sn0.7Cu-Ag-In alloy has highest value 38.2 MPa, among the four solders investigated. It is obvious that the Ag and In addition can enhance the tensile/shear properties of solder, which can be attributable to the strengthening mechanism of IMC phases such as Ag_3_Sn, InSn_4_ and In_4_Ag_9_ in the alloy matrix.

**Figure 24.**
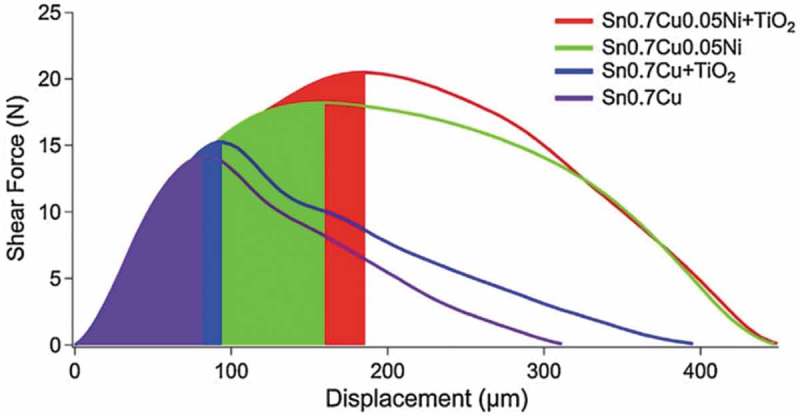
Shear force versus displacement graph for high-speed shear tests with the colored area showing the initial fracture energy of the as-soldered solder joint samples. Reproduced with permission from [72].

The growth of the interfacial layer of solder joints can be effectively inhibited by adding alloy elements or compound particles into the solder. The tensile properties of Sn-0.7Cu solder could be significantly improved by adding nano-TiO_2_ particles to the solder. When the content of nanoparticles was 1%, the UTS increased by 33.4%, and the yield limit of alloy increased by 43% [50]. As shown in Figure 24, the Ni and nano-TiO_2_ particles addition could increase the shear stress of Sn-0.7Cu solder joints to varying degrees, and the slope of the shear stress curves of Sn0.7Cu and Sn0.7Cu-TiO_2_ as reflowed are also reduced, which indicates the better fracture characteristics [72]. With 1% TiO_2_ addition, the average shear strength of Sn0.7Cu solder joint was increased by about 20% while the addition of 0.05% Ni increased that by about 28%. This strengthening effect may be attributed to the inhibition of Cu_3_Sn IMC layer by nanoparticles and the positioning of dislocations in the solder matrix. In addition, the tensile properties of Sn-0.7Cu solder joints could be improved by adding micron-sized Zn particles [45]. When the content of Zn particles was 0.2%, the UTS of composite solder joints was 41.26MPa which was increased by nearly 20%. Zn particles could make fine Cu_6_Sn_5_ particles disperse on the β-Sn matrix and play a role of dispersion strengthening, hindering the dislocation movement in the tensile process, thus improving the tensile properties of composite solder joints. However, excessive particle addition will cause the coarsening of IMC and stress concentration, resulting in crack initiation and propagation during the tensile process. Sn-0.7Cu lead-free composite solders with different content of Si_3_N_4_ were prepared by powder metallurgy [120]. The effects of different content of Si_3_N_4_ particles on the melting point temperature, hardness, shear strength and surface fracture mechanism of the solder was investigated. The results showed that when the content of Si_3_N_4_ particles exceeds 1%, the strength performance of the solder joint rose significantly.

**Figure 25.**
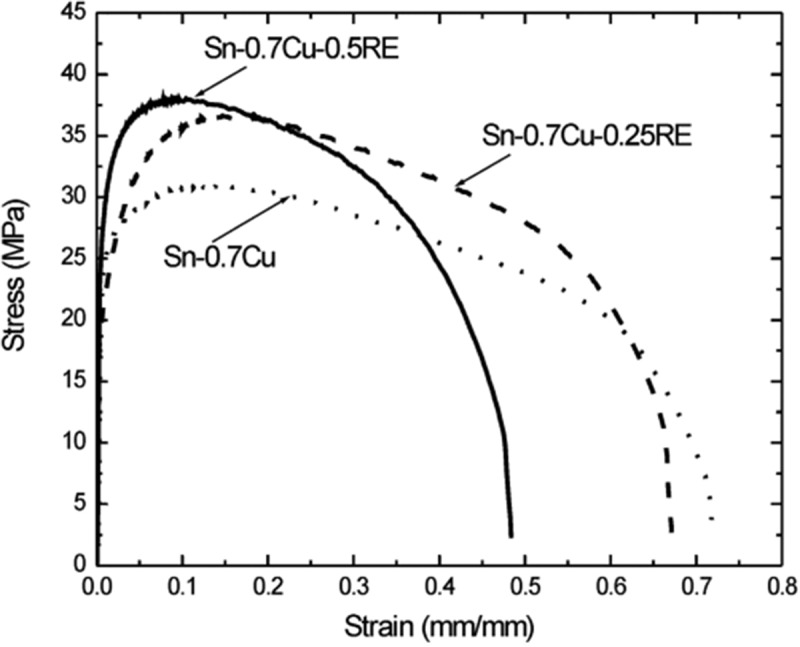
Comparative tensile stress-strain curves for Sn-0.7%Cu, Sn-0.7%Cu-0.25%RE, and Sn-0.7%Cu-0.5%RE solder alloys. Reproduced with permission from [52].

The solid solution strengthening of rare earth atoms, the grain refinement and stress concentration along the irregular shape of rare earth particles are considered to be the main factors affecting the mechanical properties of solder alloys. A number of experiments showed that the mechanical properties of lead-free solder joints can be improved by about 12% with the addition of rare earth elements (Ce, Y, La, Er), while the mechanical properties of lead-free solder joints can be improved by nearly 25% with the addition of Pr and Nd, which may be related to the distribution of Pr and Nd elements outside the arrangement of extranuclear electrons [54]. Zeng et al. [121] considered that the tensile strength of solder joints could be improved to the greatest extent when the content of Nd in Sn-0.7Cu-0.05Ni solder reached 0.05%. The addition of Nd changed the morphology of the IMC layer and made it flatter. In addition, the addition of Nd reduced the activity of Sn at the interface, inhibited the excessive growth of IMC layer at the interface, and reduced the spalling tendency of IMC layer at the solder joint interface. As shown in Figure 25, with 0.25% and 0.5% RE addition, the UTS of Sn-0.7Cu composite solder is increased by 20% and 27%, respectively [52]. On the other hand, the elongation of Sn-0.7Cu composite solder shows a decline under normal conditions.

**Figure 26.**
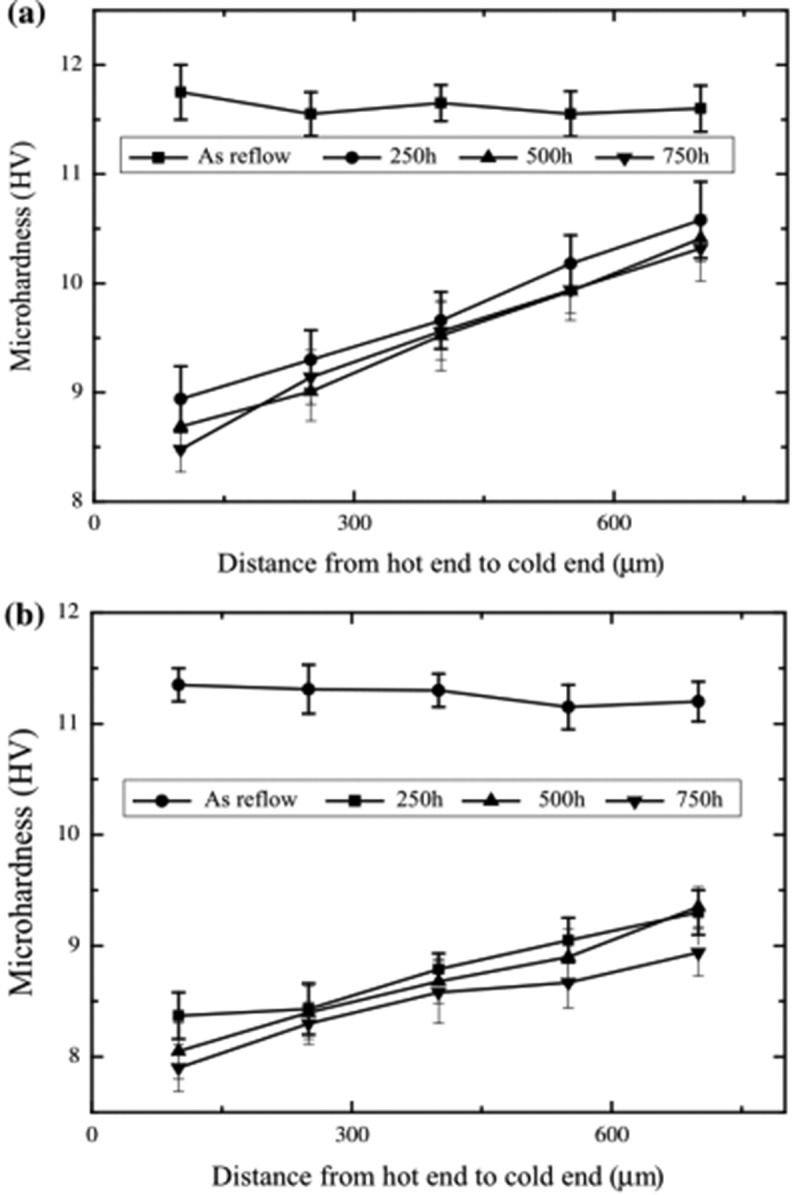
Microhardness across the solder height under a temperature gradient of 1046 °C/cm, (a) Cu/Sn/Cu solder joints, (b) Cu/Sn0.7Cu/Cu solder joints. Reproduced with permission from [124].

With the development trend of miniaturization of electronic products, the phenomenon of thermal transfer (TM) accompanied with electromigration has become one of the important factors affecting the reliability of solder joints in electronic packaging [63,122]. The accumulation and dissolution of interfacial IMCs are significantly different at both cold and hot ends due to the existence of the temperature gradient, thus affecting the mechanical properties of the solder joints. The mean microhardness of pure Sn and Sn0.7Cu solder is shown in Figure 26 [123]. Both pure Sn and Sn0.7Cu solder exhibited lower microhardness under the action of the temperature gradient. The hardness increases at a relatively constant rate from the hot end to the cold end, which means that the mechanical damage of the hot end is greater than that of the cold end. This may be due to the grain coarsening and the increase of vacancy concentration caused by high temperature at the hot end, leading to the decrease of microhardness.

Creep is the tendency of the slow permanent movement or deformation of solid materials under the influence of stress. The rate of deformation is related to material properties, loading time, loading temperature and structural stress. For some low melting point metals such as Pb and Sn, creep could occur at room temperature. The creep behaviour in Sn-Cu lead-free solder alloy is still easy to occur during service since its melting point [124]. Dislocation climbing and grain boundary sliding are the main mechanisms of creep.

**Figure 27.**
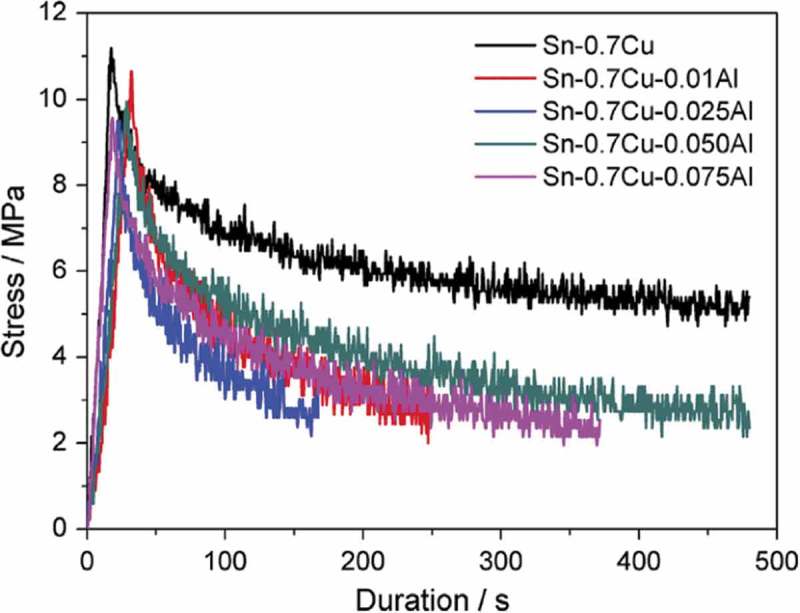
Stress relaxation curves of Sn-Cu-Al solder alloy under stress 10 MPa. Reproduced with permission from [69].

The stress-time relationship Sn-Cu-xAl solder alloy is shown in Figure 27 [69]. It can be seen that the addition of Al does not improve the creep properties of Sn-Cu alloy. The creep resistance of the alloy is guaranteed by the dislocation movement hindered by the dispersed phase. Dislocations are rearranged or annihilated in the combination of positive and negative dislocations. In the dispersion-strengthened alloys cross-slip and multiple slip occur at grain boundaries due to the effect of precipitation relative dislocation motion can reduce the creep rate. However, under the condition of grain boundary precipitation, the creep resistance of the alloy will be reduced in the poor region of the grain boundary. For Sn-Cu-Al solder alloys, Cu_6_Sn_5_ and Al_2_Cu are precipitated at grain boundaries due to the metallurgical reaction between Al and Cu, and the formation of IMC layer with continuous brittle interface promotes the formation of dilution zone. The results show that the creep resistance of Sn-Cu-A alloy is reduced by the IMC layer at the brittle interface.

After adding 2% Ag and 2% In to Sn-0.7Cu solder alloy [43], the creep rate of the solder alloy decreased by 521.0% and 200.7%, respectively. The addition of Ag and In not only resulted in the formation of intermetallic compounds Ag_3_Sn and γ-SnIn_4_, but also refined the size of β-Sn from 0.5 μm to 0.15 μm. The creep resistance of the solder alloy was improved significantly. It was found that the creep fatigue life of the solder joints containing 1% nano-Ag particles was significantly higher than that of Sn-0.7Cu and the creep activation energy was about 6.7kJ/mol higher than that of Sn-0.7Cu [125]. This attributed to the nano-Ag particles are dispersed in the β-Sn matrix, which hinders the dislocation movement during creep, thus improving the creep fatigue life of solder joints.

The creep resistance of materials has been researched from the microstructure of solder alloy or solder joints. For example, the addition of the alloy element Ag can reduce the stacking fault energy of the solder, widen the extended dislocation and prevent the edge dislocation from climbing, and the formation and movement of the vacancy can also be prevented by the dispersion strengthening of the alloy. Shi et al. [126] researched the effect of nano-Ag particles on the creep deformation of Sn-0.7Cu solder joint. The stress-strain relationship of the solder joint creep was discussed in stages and the creep constitutive equation was fitted as shown in Equations (5)–(8):

Sn-Cu solder joint constitutive equation:

(5) $$\begin{array}{rl} & \varepsilon_{1}=9.56\times 10^{24}\frac{G}{T}{\left(\frac{\tau }{G}\right)}^{6.48}\\ & \;\;\;\;\;\;\;\;\;\;\;\;\times \exp\left(-\frac{1.01\times 10^{-5}}{RT}\right)(low\;\;\;\;stress) \end{array}$$

(6) $$\begin{array}{rl} & \varepsilon_{c}=9.54\times 10^{26}\frac{G}{T}{\left(\frac{\tau }{G}\right)}^{8.73}\\ & \;\;\;\;\;\;\;\;\;\;\;\times \exp\left(-\frac{6.7\times 10^{4}}{RT}\right)(high\;\;\;\;stress) \end{array}$$

Creep constitutive equation of SnCu composite solder containing nano-Ag particles:

(7) $$\begin{array}{rl} & \varepsilon_{1}=9.83\times 10^{29}\frac{G}{T}{\left(\frac{\tau }{G}\right)}^{8.2}\\ & \;\;\;\;\;\;\;\;\;\;\;\;\times \exp\left(-\frac{1.05\times 10^{-5}}{RT}\right)(low\;\;\;\;stress) \end{array}$$

(8) $$\begin{array}{rl} & \varepsilon_{c}=4.58\times 10^{32}\frac{G}{T}{\left(\frac{\tau }{G}\right)}^{10.84}\;\;\\ & \;\;\;\;\;\;\;\;\;\;\;\;\;\times \exp\left(-\frac{7.2\times 10^{4}}{RT}\right)(high\;\;\;\;stress) \end{array}$$

where ɛ_1_ and ɛ_c_ are the strain rate, *R* is gas constant, *T* is thermodynamic temperature, *τ* is the applied shear force.

The anisotropy of alloy castings can usually be achieved by rapid solidification and directional solidification, which can improve some properties. With 0.5% Zn or Bi addition [127], the creep resistance of as-quenched melt-spun Sn-Cu composite solder alloy decreased, especially for the Sn-Cu-Bi alloy. The creep stage rapidly reaches the third stage at about 70s and cracks appear at 90s, which may be related to the high concentration vacancies obtained by rapid solidification [128].

## 7. Conclusions

Sn-Cu lead-free solders are important metal interconnection materials in electronic industry. Researchers improved their performance by alloying, particle strengthening, optimization of soldering process and development of matching soldering fluxes. However, the current development is mainly focused on the performance of the solder itself, while the reliability of joints formed after soldering is less evaluated. It is essential to understand the structure-performance relationships and potential reliability problems of Sn-Cu solders. It may also be useful to investigate new Sn-Cu based solders for specific electronic components.
